# Search for supersymmetry in a final state containing two photons and missing transverse momentum in $\varvec{\sqrt{s}}$ = 13 TeV $\varvec{pp}$ collisions at the LHC using the ATLAS detector

**DOI:** 10.1140/epjc/s10052-016-4344-x

**Published:** 2016-09-24

**Authors:** M. Aaboud, G. Aad, B. Abbott, J. Abdallah, O. Abdinov, B. Abeloos, R. Aben, O. S. AbouZeid, N. L. Abraham, H. Abramowicz, H. Abreu, R. Abreu, Y. Abulaiti, B. S. Acharya, S. Adachi, L. Adamczyk, D. L. Adams, J. Adelman, S. Adomeit, T. Adye, A. A. Affolder, T. Agatonovic-Jovin, J. Agricola, J. A. Aguilar-Saavedra, S. P. Ahlen, F. Ahmadov, G. Aielli, H. Akerstedt, T. P. A. Åkesson, A. V. Akimov, G. L. Alberghi, J. Albert, S. Albrand, M. J. Alconada Verzini, M. Aleksa, I. N. Aleksandrov, C. Alexa, G. Alexander, T. Alexopoulos, M. Alhroob, B. Ali, M. Aliev, G. Alimonti, J. Alison, S. P. Alkire, B. M. M. Allbrooke, B. W. Allen, P. P. Allport, A. Aloisio, A. Alonso, F. Alonso, C. Alpigiani, A. A. Alshehri, M. Alstaty, B. Alvarez Gonzalez, D. Álvarez Piqueras, M. G. Alviggi, B. T. Amadio, K. Amako, Y. Amaral Coutinho, C. Amelung, D. Amidei, S. P. Amor Dos Santos, A. Amorim, S. Amoroso, G. Amundsen, C. Anastopoulos, L. S. Ancu, N. Andari, T. Andeen, C. F. Anders, G. Anders, J. K. Anders, K. J. Anderson, A. Andreazza, V. Andrei, S. Angelidakis, I. Angelozzi, P. Anger, A. Angerami, F. Anghinolfi, A. V. Anisenkov, N. Anjos, A. Annovi, C. Antel, M. Antonelli, A. Antonov, F. Anulli, M. Aoki, L. Aperio Bella, G. Arabidze, Y. Arai, J. P. Araque, A. T. H. Arce, F. A. Arduh, J-F. Arguin, S. Argyropoulos, M. Arik, A. J. Armbruster, L. J. Armitage, O. Arnaez, H. Arnold, M. Arratia, O. Arslan, A. Artamonov, G. Artoni, S. Artz, S. Asai, N. Asbah, A. Ashkenazi, B. Åsman, L. Asquith, K. Assamagan, R. Astalos, M. Atkinson, N. B. Atlay, K. Augsten, G. Avolio, B. Axen, M. K. Ayoub, G. Azuelos, M. A. Baak, A. E. Baas, M. J. Baca, H. Bachacou, K. Bachas, M. Backes, M. Backhaus, P. Bagiacchi, P. Bagnaia, Y. Bai, J. T. Baines, O. K. Baker, E. M. Baldin, P. Balek, T. Balestri, F. Balli, W. K. Balunas, E. Banas, Sw. Banerjee, A. A. E. Bannoura, L. Barak, E. L. Barberio, D. Barberis, M. Barbero, T. Barillari, M-S Barisits, T. Barklow, N. Barlow, S. L. Barnes, B. M. Barnett, R. M. Barnett, Z. Barnovska-Blenessy, A. Baroncelli, G. Barone, A. J. Barr, L. Barranco Navarro, F. Barreiro, J. Barreiro Guimarães da Costa, R. Bartoldus, A. E. Barton, P. Bartos, A. Basalaev, A. Bassalat, R. L. Bates, S. J. Batista, J. R. Batley, M. Battaglia, M. Bauce, F. Bauer, H. S. Bawa, J. B. Beacham, M. D. Beattie, T. Beau, P. H. Beauchemin, P. Bechtle, H. P. Beck, K. Becker, M. Becker, M. Beckingham, C. Becot, A. J. Beddall, A. Beddall, V. A. Bednyakov, M. Bedognetti, C. P. Bee, L. J. Beemster, T. A. Beermann, M. Begel, J. K. Behr, C. Belanger-Champagne, A. S. Bell, G. Bella, L. Bellagamba, A. Bellerive, M. Bellomo, K. Belotskiy, O. Beltramello, N. L. Belyaev, O. Benary, D. Benchekroun, M. Bender, K. Bendtz, N. Benekos, Y. Benhammou, E. Benhar Noccioli, J. Benitez, D. P. Benjamin, J. R. Bensinger, S. Bentvelsen, L. Beresford, M. Beretta, D. Berge, E. Bergeaas Kuutmann, N. Berger, J. Beringer, S. Berlendis, N. R. Bernard, C. Bernius, F. U. Bernlochner, T. Berry, P. Berta, C. Bertella, G. Bertoli, F. Bertolucci, I. A. Bertram, C. Bertsche, D. Bertsche, G. J. Besjes, O. Bessidskaia Bylund, M. Bessner, N. Besson, C. Betancourt, A. Bethani, S. Bethke, A. J. Bevan, R. M. Bianchi, L. Bianchini, M. Bianco, O. Biebel, D. Biedermann, R. Bielski, N. V. Biesuz, M. Biglietti, J. Bilbao De Mendizabal, T. R. V. Billoud, H. Bilokon, M. Bindi, S. Binet, A. Bingul, C. Bini, S. Biondi, T. Bisanz, D. M. Bjergaard, C. W. Black, J. E. Black, K. M. Black, D. Blackburn, R. E. Blair, J. -B. Blanchard, T. Blazek, I. Bloch, C. Blocker, A. Blue, W. Blum, U. Blumenschein, S. Blunier, G. J. Bobbink, V. S. Bobrovnikov, S. S. Bocchetta, A. Bocci, C. Bock, M. Boehler, D. Boerner, J. A. Bogaerts, D. Bogavac, A. G. Bogdanchikov, C. Bohm, V. Boisvert, P. Bokan, T. Bold, A. S. Boldyrev, M. Bomben, M. Bona, M. Boonekamp, A. Borisov, G. Borissov, J. Bortfeldt, D. Bortoletto, V. Bortolotto, K. Bos, D. Boscherini, M. Bosman, J. D. Bossio Sola, J. Boudreau, J. Bouffard, E. V. Bouhova-Thacker, D. Boumediene, C. Bourdarios, S. K. Boutle, A. Boveia, J. Boyd, I. R. Boyko, J. Bracinik, A. Brandt, G. Brandt, O. Brandt, U. Bratzler, B. Brau, J. E. Brau, H. M. Braun, W. D. Breaden Madden, K. Brendlinger, A. J. Brennan, L. Brenner, R. Brenner, S. Bressler, T. M. Bristow, D. Britton, D. Britzger, F. M. Brochu, I. Brock, R. Brock, G. Brooijmans, T. Brooks, W. K. Brooks, J. Brosamer, E. Brost, J. H Broughton, P. A. Bruckman de Renstrom, D. Bruncko, R. Bruneliere, A. Bruni, G. Bruni, L. S. Bruni, BH Brunt, M. Bruschi, N. Bruscino, P. Bryant, L. Bryngemark, T. Buanes, Q. Buat, P. Buchholz, A. G. Buckley, I. A. Budagov, F. Buehrer, M. K. Bugge, O. Bulekov, D. Bullock, H. Burckhart, S. Burdin, C. D. Burgard, B. Burghgrave, K. Burka, S. Burke, I. Burmeister, J. T. P. Burr, E. Busato, D. Büscher, V. Büscher, P. Bussey, J. M. Butler, C. M. Buttar, J. M. Butterworth, P. Butti, W. Buttinger, A. Buzatu, A. R. Buzykaev, S. Cabrera Urbán, D. Caforio, V. M. Cairo, O. Cakir, N. Calace, P. Calafiura, A. Calandri, G. Calderini, P. Calfayan, G. Callea, L. P. Caloba, S. Calvente Lopez, D. Calvet, S. Calvet, T. P. Calvet, R. Camacho Toro, S. Camarda, P. Camarri, D. Cameron, R. Caminal Armadans, C. Camincher, S. Campana, M. Campanelli, A. Camplani, A. Campoverde, V. Canale, A. Canepa, M. Cano Bret, J. Cantero, T. Cao, M. D. M. Capeans Garrido, I. Caprini, M. Caprini, M. Capua, R. M. Carbone, R. Cardarelli, F. Cardillo, I. Carli, T. Carli, G. Carlino, L. Carminati, S. Caron, E. Carquin, G. D. Carrillo-Montoya, J. R. Carter, J. Carvalho, D. Casadei, M. P. Casado, M. Casolino, D. W. Casper, E. Castaneda-Miranda, R. Castelijn, A. Castelli, V. Castillo Gimenez, N. F. Castro, A. Catinaccio, J. R. Catmore, A. Cattai, J. Caudron, V. Cavaliere, E. Cavallaro, D. Cavalli, M. Cavalli-Sforza, V. Cavasinni, F. Ceradini, L. Cerda Alberich, B. C. Cerio, A. S. Cerqueira, A. Cerri, L. Cerrito, F. Cerutti, M. Cerv, A. Cervelli, S. A. Cetin, A. Chafaq, D. Chakraborty, S. K. Chan, Y. L. Chan, P. Chang, J. D. Chapman, D. G. Charlton, A. Chatterjee, C. C. Chau, C. A. Chavez Barajas, S. Che, S. Cheatham, A. Chegwidden, S. Chekanov, S. V. Chekulaev, G. A. Chelkov, M. A. Chelstowska, C. Chen, H. Chen, K. Chen, S. Chen, S. Chen, X. Chen, Y. Chen, H. C. Cheng, H. J Cheng, Y. Cheng, A. Cheplakov, E. Cheremushkina, R. Cherkaoui El Moursli, V. Chernyatin, E. Cheu, L. Chevalier, V. Chiarella, G. Chiarelli, G. Chiodini, A. S. Chisholm, A. Chitan, M. V. Chizhov, K. Choi, A. R. Chomont, S. Chouridou, B. K. B. Chow, V. Christodoulou, D. Chromek-Burckhart, J. Chudoba, A. J. Chuinard, J. J. Chwastowski, L. Chytka, G. Ciapetti, A. K. Ciftci, D. Cinca, V. Cindro, I. A. Cioara, C. Ciocca, A. Ciocio, F. Cirotto, Z. H. Citron, M. Citterio, M. Ciubancan, A. Clark, B. L. Clark, M. R. Clark, P. J. Clark, R. N. Clarke, C. Clement, Y. Coadou, M. Cobal, A. Coccaro, J. Cochran, L. Colasurdo, B. Cole, A. P. Colijn, J. Collot, T. Colombo, G. Compostella, P. Conde Muiño, E. Coniavitis, S. H. Connell, I. A. Connelly, V. Consorti, S. Constantinescu, G. Conti, F. Conventi, M. Cooke, B. D. Cooper, A. M. Cooper-Sarkar, K. J. R. Cormier, T. Cornelissen, M. Corradi, F. Corriveau, A. Corso-Radu, A. Cortes-Gonzalez, G. Cortiana, G. Costa, M. J. Costa, D. Costanzo, G. Cottin, G. Cowan, B. E. Cox, K. Cranmer, S. J. Crawley, G. Cree, S. Crépé-Renaudin, F. Crescioli, W. A. Cribbs, M. Crispin Ortuzar, M. Cristinziani, V. Croft, G. Crosetti, A. Cueto, T. Cuhadar Donszelmann, J. Cummings, M. Curatolo, J. Cúth, H. Czirr, P. Czodrowski, G. D’amen, S. D’Auria, M. D’Onofrio, M. J. Da Cunha Sargedas De Sousa, C. Da Via, W. Dabrowski, T. Dado, T. Dai, O. Dale, F. Dallaire, C. Dallapiccola, M. Dam, J. R. Dandoy, N. P. Dang, A. C. Daniells, N. S. Dann, M. Danninger, M. Dano Hoffmann, V. Dao, G. Darbo, S. Darmora, J. Dassoulas, A. Dattagupta, W. Davey, C. David, T. Davidek, M. Davies, P. Davison, E. Dawe, I. Dawson, K. De, R. de Asmundis, A. De Benedetti, S. De Castro, S. De Cecco, N. De Groot, P. de Jong, H. De la Torre, F. De Lorenzi, A. De Maria, D. De Pedis, A. De Salvo, U. De Sanctis, A. De Santo, J. B. De Vivie De Regie, W. J. Dearnaley, R. Debbe, C. Debenedetti, D. V. Dedovich, N. Dehghanian, I. Deigaard, M. Del Gaudio, J. Del Peso, T. Del Prete, D. Delgove, F. Deliot, C. M. Delitzsch, A. Dell’Acqua, L. Dell’Asta, M. Dell’Orso, M. Della Pietra, D. della Volpe, M. Delmastro, P. A. Delsart, D. A. DeMarco, S. Demers, M. Demichev, A. Demilly, S. P. Denisov, D. Denysiuk, D. Derendarz, J. E. Derkaoui, F. Derue, P. Dervan, K. Desch, C. Deterre, K. Dette, P. O. Deviveiros, A. Dewhurst, S. Dhaliwal, A. Di Ciaccio, L. Di Ciaccio, W. K. Di Clemente, C. Di Donato, A. Di Girolamo, B. Di Girolamo, B. Di Micco, R. Di Nardo, A. Di Simone, R. Di Sipio, D. Di Valentino, C. Diaconu, M. Diamond, F. A. Dias, M. A. Diaz, E. B. Diehl, J. Dietrich, S. Díez Cornell, A. Dimitrievska, J. Dingfelder, P. Dita, S. Dita, F. Dittus, F. Djama, T. Djobava, J. I. Djuvsland, M. A. B. do Vale, D. Dobos, M. Dobre, C. Doglioni, J. Dolejsi, Z. Dolezal, M. Donadelli, S. Donati, P. Dondero, J. Donini, J. Dopke, A. Doria, M. T. Dova, A. T. Doyle, E. Drechsler, M. Dris, Y. Du, J. Duarte-Campderros, E. Duchovni, G. Duckeck, O. A. Ducu, D. Duda, A. Dudarev, A. Chr. Dudder, E. M. Duffield, L. Duflot, M. Dührssen, M. Dumancic, M. Dunford, H. Duran Yildiz, M. Düren, A. Durglishvili, D. Duschinger, B. Dutta, M. Dyndal, C. Eckardt, K. M. Ecker, R. C. Edgar, N. C. Edwards, T. Eifert, G. Eigen, K. Einsweiler, T. Ekelof, M. El Kacimi, V. Ellajosyula, M. Ellert, S. Elles, F. Ellinghaus, A. A. Elliot, N. Ellis, J. Elmsheuser, M. Elsing, D. Emeliyanov, Y. Enari, O. C. Endner, J. S. Ennis, J. Erdmann, A. Ereditato, G. Ernis, J. Ernst, M. Ernst, S. Errede, E. Ertel, M. Escalier, H. Esch, C. Escobar, B. Esposito, A. I. Etienvre, E. Etzion, H. Evans, A. Ezhilov, M. Ezzi, F. Fabbri, L. Fabbri, G. Facini, R. M. Fakhrutdinov, S. Falciano, R. J. Falla, J. Faltova, Y. Fang, M. Fanti, A. Farbin, A. Farilla, C. Farina, E. M. Farina, T. Farooque, S. Farrell, S. M. Farrington, P. Farthouat, F. Fassi, P. Fassnacht, D. Fassouliotis, M. Faucci Giannelli, A. Favareto, W. J. Fawcett, L. Fayard, O. L. Fedin, W. Fedorko, S. Feigl, L. Feligioni, C. Feng, E. J. Feng, H. Feng, A. B. Fenyuk, L. Feremenga, P. Fernandez Martinez, S. Fernandez Perez, J. Ferrando, A. Ferrari, P. Ferrari, R. Ferrari, D. E. Ferreira de Lima, A. Ferrer, D. Ferrere, C. Ferretti, A. Ferretto Parodi, F. Fiedler, A. Filipčič, M. Filipuzzi, F. Filthaut, M. Fincke-Keeler, K. D. Finelli, M. C. N. Fiolhais, L. Fiorini, A. Firan, A. Fischer, C. Fischer, J. Fischer, W. C. Fisher, N. Flaschel, I. Fleck, P. Fleischmann, G. T. Fletcher, R. R. M. Fletcher, T. Flick, L. R. Flores Castillo, M. J. Flowerdew, G. T. Forcolin, A. Formica, A. Forti, A. G. Foster, D. Fournier, H. Fox, S. Fracchia, P. Francavilla, M. Franchini, D. Francis, L. Franconi, M. Franklin, M. Frate, M. Fraternali, D. Freeborn, S. M. Fressard-Batraneanu, F. Friedrich, D. Froidevaux, J. A. Frost, C. Fukunaga, E. Fullana Torregrosa, T. Fusayasu, J. Fuster, C. Gabaldon, O. Gabizon, A. Gabrielli, A. Gabrielli, G. P. Gach, S. Gadatsch, S. Gadomski, G. Gagliardi, L. G. Gagnon, P. Gagnon, C. Galea, B. Galhardo, E. J. Gallas, B. J. Gallop, P. Gallus, G. Galster, K. K. Gan, J. Gao, Y. Gao, Y. S. Gao, F. M. Garay Walls, C. García, J. E. García Navarro, M. Garcia-Sciveres, R. W. Gardner, N. Garelli, V. Garonne, A. Gascon Bravo, K. Gasnikova, C. Gatti, A. Gaudiello, G. Gaudio, L. Gauthier, I. L. Gavrilenko, C. Gay, G. Gaycken, E. N. Gazis, Z. Gecse, C. N. P. Gee, Ch. Geich-Gimbel, M. Geisen, M. P. Geisler, K. Gellerstedt, C. Gemme, M. H. Genest, C. Geng, S. Gentile, C. Gentsos, S. George, D. Gerbaudo, A. Gershon, S. Ghasemi, M. Ghneimat, B. Giacobbe, S. Giagu, P. Giannetti, B. Gibbard, S. M. Gibson, M. Gignac, M. Gilchriese, T. P. S. Gillam, D. Gillberg, G. Gilles, D. M. Gingrich, N. Giokaris, M. P. Giordani, F. M. Giorgi, F. M. Giorgi, P. F. Giraud, P. Giromini, D. Giugni, F. Giuli, C. Giuliani, M. Giulini, B. K. Gjelsten, S. Gkaitatzis, I. Gkialas, E. L. Gkougkousis, L. K. Gladilin, C. Glasman, J. Glatzer, P. C. F. Glaysher, A. Glazov, M. Goblirsch-Kolb, J. Godlewski, S. Goldfarb, T. Golling, D. Golubkov, A. Gomes, R. Gonçalo, J. Goncalves Pinto Firmino Da Costa, G. Gonella, L. Gonella, A. Gongadze, S. González de la Hoz, G. Gonzalez Parra, S. Gonzalez-Sevilla, L. Goossens, P. A. Gorbounov, H. A. Gordon, I. Gorelov, B. Gorini, E. Gorini, A. Gorišek, E. Gornicki, A. T. Goshaw, C. Gössling, M. I. Gostkin, C. R. Goudet, D. Goujdami, A. G. Goussiou, N. Govender, E. Gozani, L. Graber, I. Grabowska-Bold, P. O. J. Gradin, P. Grafström, J. Gramling, E. Gramstad, S. Grancagnolo, V. Gratchev, P. M. Gravila, H. M. Gray, E. Graziani, Z. D. Greenwood, C. Grefe, K. Gregersen, I. M. Gregor, P. Grenier, K. Grevtsov, J. Griffiths, A. A. Grillo, K. Grimm, S. Grinstein, Ph. Gris, J. -F. Grivaz, S. Groh, J. P. Grohs, E. Gross, J. Grosse-Knetter, G. C. Grossi, Z. J. Grout, L. Guan, W. Guan, J. Guenther, F. Guescini, D. Guest, O. Gueta, E. Guido, T. Guillemin, S. Guindon, U. Gul, C. Gumpert, J. Guo, Y. Guo, R. Gupta, S. Gupta, G. Gustavino, P. Gutierrez, N. G. Gutierrez Ortiz, C. Gutschow, C. Guyot, C. Gwenlan, C. B. Gwilliam, A. Haas, C. Haber, H. K. Hadavand, N. Haddad, A. Hadef, S. Hageböck, M. Hagihara, Z. Hajduk, H. Hakobyan, M. Haleem, J. Haley, G. Halladjian, G. D. Hallewell, K. Hamacher, P. Hamal, K. Hamano, A. Hamilton, G. N. Hamity, P. G. Hamnett, L. Han, K. Hanagaki, K. Hanawa, M. Hance, B. Haney, P. Hanke, R. Hanna, J. B. Hansen, J. D. Hansen, M. C. Hansen, P. H. Hansen, K. Hara, A. S. Hard, T. Harenberg, F. Hariri, S. Harkusha, R. D. Harrington, P. F. Harrison, F. Hartjes, N. M. Hartmann, M. Hasegawa, Y. Hasegawa, A. Hasib, S. Hassani, S. Haug, R. Hauser, L. Hauswald, M. Havranek, C. M. Hawkes, R. J. Hawkings, D. Hayakawa, D. Hayden, C. P. Hays, J. M. Hays, H. S. Hayward, S. J. Haywood, S. J. Head, T. Heck, V. Hedberg, L. Heelan, S. Heim, T. Heim, B. Heinemann, J. J. Heinrich, L. Heinrich, C. Heinz, J. Hejbal, L. Helary, S. Hellman, C. Helsens, J. Henderson, R. C. W. Henderson, Y. Heng, S. Henkelmann, A. M. Henriques Correia, S. Henrot-Versille, G. H. Herbert, H. Herde, V. Herget, Y. Hernández Jiménez, G. Herten, R. Hertenberger, L. Hervas, G. G. Hesketh, N. P. Hessey, J. W. Hetherly, R. Hickling, E. Higón-Rodriguez, E. Hill, J. C. Hill, K. H. Hiller, S. J. Hillier, I. Hinchliffe, E. Hines, R. R. Hinman, M. Hirose, D. Hirschbuehl, J. Hobbs, N. Hod, M. C. Hodgkinson, P. Hodgson, A. Hoecker, M. R. Hoeferkamp, F. Hoenig, D. Hohn, T. R. Holmes, M. Homann, T. Honda, T. M. Hong, B. H. Hooberman, W. H. Hopkins, Y. Horii, A. J. Horton, J-Y. Hostachy, S. Hou, A. Hoummada, J. Howarth, J. Hoya, M. Hrabovsky, I. Hristova, J. Hrivnac, T. Hryn’ova, A. Hrynevich, C. Hsu, P. J. Hsu, S. -C. Hsu, Q. Hu, S. Hu, Y. Huang, Z. Hubacek, F. Hubaut, F. Huegging, T. B. Huffman, E. W. Hughes, G. Hughes, M. Huhtinen, P. Huo, N. Huseynov, J. Huston, J. Huth, G. Iacobucci, G. Iakovidis, I. Ibragimov, L. Iconomidou-Fayard, E. Ideal, Z. Idrissi, P. Iengo, O. Igonkina, T. Iizawa, Y. Ikegami, M. Ikeno, Y. Ilchenko, D. Iliadis, N. Ilic, T. Ince, G. Introzzi, P. Ioannou, M. Iodice, K. Iordanidou, V. Ippolito, N. Ishijima, M. Ishino, M. Ishitsuka, R. Ishmukhametov, C. Issever, S. Istin, F. Ito, J. M. Iturbe Ponce, R. Iuppa, W. Iwanski, H. Iwasaki, J. M. Izen, V. Izzo, S. Jabbar, B. Jackson, P. Jackson, V. Jain, K. B. Jakobi, K. Jakobs, S. Jakobsen, T. Jakoubek, D. O. Jamin, D. K. Jana, R. Jansky, J. Janssen, M. Janus, G. Jarlskog, N. Javadov, T. Javůrek, F. Jeanneau, L. Jeanty, G. -Y. Jeng, D. Jennens, P. Jenni, C. Jeske, S. Jézéquel, H. Ji, J. Jia, H. Jiang, Y. Jiang, S. Jiggins, J. Jimenez Pena, S. Jin, A. Jinaru, O. Jinnouchi, H. Jivan, P. Johansson, K. A. Johns, W. J. Johnson, K. Jon-And, G. Jones, R. W. L. Jones, S. Jones, T. J. Jones, J. Jongmanns, P. M. Jorge, J. Jovicevic, X. Ju, A. Juste Rozas, M. K. Köhler, A. Kaczmarska, M. Kado, H. Kagan, M. Kagan, S. J. Kahn, T. Kaji, E. Kajomovitz, C. W. Kalderon, A. Kaluza, S. Kama, A. Kamenshchikov, N. Kanaya, S. Kaneti, L. Kanjir, V. A. Kantserov, J. Kanzaki, B. Kaplan, L. S. Kaplan, A. Kapliy, D. Kar, K. Karakostas, A. Karamaoun, N. Karastathis, M. J. Kareem, E. Karentzos, M. Karnevskiy, S. N. Karpov, Z. M. Karpova, K. Karthik, V. Kartvelishvili, A. N. Karyukhin, K. Kasahara, L. Kashif, R. D. Kass, A. Kastanas, Y. Kataoka, C. Kato, A. Katre, J. Katzy, K. Kawagoe, T. Kawamoto, G. Kawamura, V. F. Kazanin, R. Keeler, R. Kehoe, J. S. Keller, J. J. Kempster, K Kentaro, H. Keoshkerian, O. Kepka, B. P. Kerševan, S. Kersten, R. A. Keyes, M. Khader, F. Khalil-zada, A. Khanov, A. G. Kharlamov, T. Kharlamova, T. J. Khoo, V. Khovanskiy, E. Khramov, J. Khubua, S. Kido, C. R. Kilby, H. Y. Kim, S. H. Kim, Y. K. Kim, N. Kimura, O. M. Kind, B. T. King, M. King, J. Kirk, A. E. Kiryunin, T. Kishimoto, D. Kisielewska, F. Kiss, K. Kiuchi, O. Kivernyk, E. Kladiva, M. H. Klein, M. Klein, U. Klein, K. Kleinknecht, P. Klimek, A. Klimentov, R. Klingenberg, J. A. Klinger, T. Klioutchnikova, E. -E. Kluge, P. Kluit, S. Kluth, J. Knapik, E. Kneringer, E. B. F. G. Knoops, A. Knue, A. Kobayashi, D. Kobayashi, T. Kobayashi, M. Kobel, M. Kocian, P. Kodys, N. M. Koehler, T. Koffas, E. Koffeman, T. Koi, H. Kolanoski, M. Kolb, I. Koletsou, A. A. Komar, Y. Komori, T. Kondo, N. Kondrashova, K. Köneke, A. C. König, T. Kono, R. Konoplich, N. Konstantinidis, R. Kopeliansky, S. Koperny, L. Köpke, A. K. Kopp, K. Korcyl, K. Kordas, A. Korn, A. A. Korol, I. Korolkov, E. V. Korolkova, O. Kortner, S. Kortner, T. Kosek, V. V. Kostyukhin, A. Kotwal, A. Kourkoumeli-Charalampidi, C. Kourkoumelis, V. Kouskoura, A. B. Kowalewska, R. Kowalewski, T. Z. Kowalski, C. Kozakai, W. Kozanecki, A. S. Kozhin, V. A. Kramarenko, G. Kramberger, D. Krasnopevtsev, M. W. Krasny, A. Krasznahorkay, A. Kravchenko, M. Kretz, J. Kretzschmar, K. Kreutzfeldt, P. Krieger, K. Krizka, K. Kroeninger, H. Kroha, J. Kroll, J. Kroseberg, J. Krstic, U. Kruchonak, H. Krüger, N. Krumnack, A. Kruse, M. C. Kruse, M. Kruskal, T. Kubota, H. Kucuk, S. Kuday, J. T. Kuechler, S. Kuehn, A. Kugel, F. Kuger, A. Kuhl, T. Kuhl, V. Kukhtin, R. Kukla, Y. Kulchitsky, S. Kuleshov, M. Kuna, T. Kunigo, A. Kupco, H. Kurashige, Y. A. Kurochkin, V. Kus, E. S. Kuwertz, M. Kuze, J. Kvita, T. Kwan, D. Kyriazopoulos, A. La Rosa, J. L. La Rosa Navarro, L. La Rotonda, C. Lacasta, F. Lacava, J. Lacey, H. Lacker, D. Lacour, V. R. Lacuesta, E. Ladygin, R. Lafaye, B. Laforge, T. Lagouri, S. Lai, S. Lammers, W. Lampl, E. Lançon, U. Landgraf, M. P. J. Landon, M. C. Lanfermann, V. S. Lang, J. C. Lange, A. J. Lankford, F. Lanni, K. Lantzsch, A. Lanza, S. Laplace, C. Lapoire, J. F. Laporte, T. Lari, F. Lasagni Manghi, M. Lassnig, P. Laurelli, W. Lavrijsen, A. T. Law, P. Laycock, T. Lazovich, M. Lazzaroni, B. Le, O. Le Dortz, E. Le Guirriec, E. P. Le Quilleuc, M. LeBlanc, T. LeCompte, F. Ledroit-Guillon, C. A. Lee, S. C. Lee, L. Lee, B. Lefebvre, G. Lefebvre, M. Lefebvre, F. Legger, C. Leggett, A. Lehan, G. Lehmann Miotto, X. Lei, W. A. Leight, A. Leisos, A. G. Leister, M. A. L. Leite, R. Leitner, D. Lellouch, B. Lemmer, K. J. C. Leney, T. Lenz, B. Lenzi, R. Leone, S. Leone, C. Leonidopoulos, S. Leontsinis, G. Lerner, C. Leroy, A. A. J. Lesage, C. G. Lester, M. Levchenko, J. Levêque, D. Levin, L. J. Levinson, M. Levy, D. Lewis, A. M. Leyko, M. Leyton, B. Li, C. Li, H. Li, H. L. Li, L. Li, L. Li, Q. Li, S. Li, X. Li, Y. Li, Z. Liang, B. Liberti, A. Liblong, P. Lichard, K. Lie, J. Liebal, W. Liebig, A. Limosani, S. C. Lin, T. H. Lin, B. E. Lindquist, A. E. Lionti, E. Lipeles, A. Lipniacka, M. Lisovyi, T. M. Liss, A. Lister, A. M. Litke, B. Liu, D. Liu, H. Liu, H. Liu, J. Liu, J. B. Liu, K. Liu, L. Liu, M. Liu, M. Liu, Y. L. Liu, Y. Liu, M. Livan, A. Lleres, J. Llorente Merino, S. L. Lloyd, F. Lo Sterzo, E. M. Lobodzinska, P. Loch, W. S. Lockman, F. K. Loebinger, A. E. Loevschall-Jensen, K. M. Loew, A. Loginov, T. Lohse, K. Lohwasser, M. Lokajicek, B. A. Long, J. D. Long, R. E. Long, L. Longo, K. A. Looper, J. A. López, D. Lopez Mateos, B. Lopez Paredes, I. Lopez Paz, A. Lopez Solis, J. Lorenz, N. Lorenzo Martinez, M. Losada, P. J. Lösel, X. Lou, A. Lounis, J. Love, P. A. Love, H. Lu, N. Lu, H. J. Lubatti, C. Luci, A. Lucotte, C. Luedtke, F. Luehring, W. Lukas, L. Luminari, O. Lundberg, B. Lund-Jensen, P. M. Luzi, D. Lynn, R. Lysak, E. Lytken, V. Lyubushkin, H. Ma, L. L. Ma, Y. Ma, G. Maccarrone, A. Macchiolo, C. M. Macdonald, B. Maček, J. Machado Miguens, D. Madaffari, R. Madar, H. J. Maddocks, W. F. Mader, A. Madsen, J. Maeda, S. Maeland, T. Maeno, A. Maevskiy, E. Magradze, J. Mahlstedt, C. Maiani, C. Maidantchik, A. A. Maier, T. Maier, A. Maio, S. Majewski, Y. Makida, N. Makovec, B. Malaescu, Pa. Malecki, V. P. Maleev, F. Malek, U. Mallik, D. Malon, C. Malone, C. Malone, S. Maltezos, S. Malyukov, J. Mamuzic, G. Mancini, L. Mandelli, I. Mandić, J. Maneira, L. Manhaes de Andrade Filho, J. Manjarres Ramos, A. Mann, A. Manousos, B. Mansoulie, J. D. Mansour, R. Mantifel, M. Mantoani, S. Manzoni, L. Mapelli, G. Marceca, L. March, G. Marchiori, M. Marcisovsky, M. Marjanovic, D. E. Marley, F. Marroquim, S. P. Marsden, Z. Marshall, S. Marti-Garcia, B. Martin, T. A. Martin, V. J. Martin, B. Martin dit Latour, M. Martinez, V. I. Martinez Outschoorn, S. Martin-Haugh, V. S. Martoiu, A. C. Martyniuk, M. Marx, A. Marzin, L. Masetti, T. Mashimo, R. Mashinistov, J. Masik, A. L. Maslennikov, I. Massa, L. Massa, P. Mastrandrea, A. Mastroberardino, T. Masubuchi, P. Mättig, J. Mattmann, J. Maurer, S. J. Maxfield, D. A. Maximov, R. Mazini, S. M. Mazza, N. C. Mc Fadden, G. Mc Goldrick, S. P. Mc Kee, A. McCarn, R. L. McCarthy, T. G. McCarthy, L. I. McClymont, E. F. McDonald, J. A. Mcfayden, G. Mchedlidze, S. J. McMahon, R. A. McPherson, M. Medinnis, S. Meehan, S. Mehlhase, A. Mehta, K. Meier, C. Meineck, B. Meirose, D. Melini, B. R. Mellado Garcia, M. Melo, F. Meloni, A. Mengarelli, S. Menke, E. Meoni, S. Mergelmeyer, P. Mermod, L. Merola, C. Meroni, F. S. Merritt, A. Messina, J. Metcalfe, A. S. Mete, C. Meyer, C. Meyer, J-P. Meyer, J. Meyer, H. Meyer Zu Theenhausen, F. Miano, R. P. Middleton, S. Miglioranzi, L. Mijović, G. Mikenberg, M. Mikestikova, M. Mikuž, M. Milesi, A. Milic, D. W. Miller, C. Mills, A. Milov, D. A. Milstead, A. A. Minaenko, Y. Minami, I. A. Minashvili, A. I. Mincer, B. Mindur, M. Mineev, Y. Minegishi, Y. Ming, L. M. Mir, K. P. Mistry, T. Mitani, J. Mitrevski, V. A. Mitsou, A. Miucci, P. S. Miyagawa, J. U. Mjörnmark, M. Mlynarikova, T. Moa, K. Mochizuki, S. Mohapatra, S. Molander, R. Moles-Valls, R. Monden, M. C. Mondragon, K. Mönig, J. Monk, E. Monnier, A. Montalbano, J. Montejo Berlingen, F. Monticelli, S. Monzani, R. W. Moore, N. Morange, D. Moreno, M. Moreno Llácer, P. Morettini, S. Morgenstern, D. Mori, T. Mori, M. Morii, M. Morinaga, V. Morisbak, S. Moritz, A. K. Morley, G. Mornacchi, J. D. Morris, S. S. Mortensen, L. Morvaj, M. Mosidze, J. Moss, K. Motohashi, R. Mount, E. Mountricha, E. J. W. Moyse, S. Muanza, R. D. Mudd, F. Mueller, J. Mueller, R. S. P. Mueller, T. Mueller, D. Muenstermann, P. Mullen, G. A. Mullier, F. J. Munoz Sanchez, J. A. Murillo Quijada, W. J. Murray, H. Musheghyan, M. Muškinja, A. G. Myagkov, M. Myska, B. P. Nachman, O. Nackenhorst, K. Nagai, R. Nagai, K. Nagano, Y. Nagasaka, K. Nagata, M. Nagel, E. Nagy, A. M. Nairz, Y. Nakahama, K. Nakamura, T. Nakamura, I. Nakano, H. Namasivayam, R. F. Naranjo Garcia, R. Narayan, D. I. Narrias Villar, I. Naryshkin, T. Naumann, G. Navarro, R. Nayyar, H. A. Neal, P. Yu. Nechaeva, T. J. Neep, A. Negri, M. Negrini, S. Nektarijevic, C. Nellist, A. Nelson, S. Nemecek, P. Nemethy, A. A. Nepomuceno, M. Nessi, M. S. Neubauer, M. Neumann, R. M. Neves, P. Nevski, P. R. Newman, D. H. Nguyen, T. Nguyen Manh, R. B. Nickerson, R. Nicolaidou, J. Nielsen, A. Nikiforov, V. Nikolaenko, I. Nikolic-Audit, K. Nikolopoulos, J. K. Nilsen, P. Nilsson, Y. Ninomiya, A. Nisati, R. Nisius, T. Nobe, M. Nomachi, I. Nomidis, T. Nooney, S. Norberg, M. Nordberg, N. Norjoharuddeen, O. Novgorodova, S. Nowak, M. Nozaki, L. Nozka, K. Ntekas, E. Nurse, F. Nuti, F. O’grady, D. C. O’Neil, A. A. O’Rourke, V. O’Shea, F. G. Oakham, H. Oberlack, T. Obermann, J. Ocariz, A. Ochi, I. Ochoa, J. P. Ochoa-Ricoux, S. Oda, S. Odaka, H. Ogren, A. Oh, S. H. Oh, C. C. Ohm, H. Ohman, H. Oide, H. Okawa, Y. Okumura, T. Okuyama, A. Olariu, L. F. Oleiro Seabra, S. A. Olivares Pino, D. Oliveira Damazio, A. Olszewski, J. Olszowska, A. Onofre, K. Onogi, P. U. E. Onyisi, M. J. Oreglia, Y. Oren, D. Orestano, N. Orlando, R. S. Orr, B. Osculati, R. Ospanov, G. Otero y Garzon, H. Otono, M. Ouchrif, F. Ould-Saada, A. Ouraou, K. P. Oussoren, Q. Ouyang, M. Owen, R. E. Owen, V. E. Ozcan, N. Ozturk, K. Pachal, A. Pacheco Pages, L. Pacheco Rodriguez, C. Padilla Aranda, M. Pagáčová, S. Pagan Griso, M. Paganini, F. Paige, P. Pais, K. Pajchel, G. Palacino, S. Palazzo, S. Palestini, M. Palka, D. Pallin, E. St. Panagiotopoulou, C. E. Pandini, J. G. Panduro Vazquez, P. Pani, S. Panitkin, D. Pantea, L. Paolozzi, Th. D. Papadopoulou, K. Papageorgiou, A. Paramonov, D. Paredes Hernandez, A. J. Parker, M. A. Parker, K. A. Parker, F. Parodi, J. A. Parsons, U. Parzefall, V. R. Pascuzzi, E. Pasqualucci, S. Passaggio, Fr. Pastore, G. Pásztor, S. Pataraia, J. R. Pater, T. Pauly, J. Pearce, B. Pearson, L. E. Pedersen, M. Pedersen, S. Pedraza Lopez, R. Pedro, S. V. Peleganchuk, O. Penc, C. Peng, H. Peng, J. Penwell, B. S. Peralva, M. M. Perego, D. V. Perepelitsa, E. Perez Codina, L. Perini, H. Pernegger, S. Perrella, R. Peschke, V. D. Peshekhonov, K. Peters, R. F. Y. Peters, B. A. Petersen, T. C. Petersen, E. Petit, A. Petridis, C. Petridou, P. Petroff, E. Petrolo, M. Petrov, F. Petrucci, N. E. Pettersson, A. Peyaud, R. Pezoa, P. W. Phillips, G. Piacquadio, E. Pianori, A. Picazio, E. Piccaro, M. Piccinini, M. A. Pickering, R. Piegaia, J. E. Pilcher, A. D. Pilkington, A. W. J. Pin, M. Pinamonti, J. L. Pinfold, A. Pingel, S. Pires, H. Pirumov, M. Pitt, L. Plazak, M. -A. Pleier, V. Pleskot, E. Plotnikova, P. Plucinski, D. Pluth, R. Poettgen, L. Poggioli, D. Pohl, G. Polesello, A. Poley, A. Policicchio, R. Polifka, A. Polini, C. S. Pollard, V. Polychronakos, K. Pommès, L. Pontecorvo, B. G. Pope, G. A. Popeneciu, A. Poppleton, S. Pospisil, K. Potamianos, I. N. Potrap, C. J. Potter, C. T. Potter, G. Poulard, J. Poveda, V. Pozdnyakov, M. E. Pozo Astigarraga, P. Pralavorio, A. Pranko, S. Prell, D. Price, L. E. Price, M. Primavera, S. Prince, K. Prokofiev, F. Prokoshin, S. Protopopescu, J. Proudfoot, M. Przybycien, D. Puddu, M. Purohit, P. Puzo, J. Qian, G. Qin, Y. Qin, A. Quadt, W. B. Quayle, M. Queitsch-Maitland, D. Quilty, S. Raddum, V. Radeka, V. Radescu, S. K. Radhakrishnan, P. Radloff, P. Rados, F. Ragusa, G. Rahal, J. A. Raine, S. Rajagopalan, M. Rammensee, C. Rangel-Smith, M. G. Ratti, F. Rauscher, S. Rave, T. Ravenscroft, I. Ravinovich, M. Raymond, A. L. Read, N. P. Readioff, M. Reale, D. M. Rebuzzi, A. Redelbach, G. Redlinger, R. Reece, K. Reeves, L. Rehnisch, J. Reichert, A. Reiss, C. Rembser, H. Ren, M. Rescigno, S. Resconi, O. L. Rezanova, P. Reznicek, R. Rezvani, R. Richter, S. Richter, E. Richter-Was, O. Ricken, M. Ridel, P. Rieck, C. J. Riegel, J. Rieger, O. Rifki, M. Rijssenbeek, A. Rimoldi, M. Rimoldi, L. Rinaldi, B. Ristić, E. Ritsch, I. Riu, F. Rizatdinova, E. Rizvi, C. Rizzi, S. H. Robertson, A. Robichaud-Veronneau, D. Robinson, J. E. M. Robinson, A. Robson, C. Roda, Y. Rodina, A. Rodriguez Perez, D. Rodriguez Rodriguez, S. Roe, C. S. Rogan, O. Røhne, A. Romaniouk, M. Romano, S. M. Romano Saez, E. Romero Adam, N. Rompotis, M. Ronzani, L. Roos, E. Ros, S. Rosati, K. Rosbach, P. Rose, N. -A. Rosien, V. Rossetti, E. Rossi, L. P. Rossi, J. H. N. Rosten, R. Rosten, M. Rotaru, I. Roth, J. Rothberg, D. Rousseau, A. Rozanov, Y. Rozen, X. Ruan, F. Rubbo, M. S. Rudolph, F. Rühr, A. Ruiz-Martinez, Z. Rurikova, N. A. Rusakovich, A. Ruschke, H. L. Russell, J. P. Rutherfoord, N. Ruthmann, Y. F. Ryabov, M. Rybar, G. Rybkin, S. Ryu, A. Ryzhov, G. F. Rzehorz, A. F. Saavedra, G. Sabato, S. Sacerdoti, H. F-W. Sadrozinski, R. Sadykov, F. Safai Tehrani, P. Saha, M. Sahinsoy, M. Saimpert, T. Saito, H. Sakamoto, Y. Sakurai, G. Salamanna, A. Salamon, J. E. Salazar Loyola, D. Salek, P. H. Sales De Bruin, D. Salihagic, A. Salnikov, J. Salt, D. Salvatore, F. Salvatore, A. Salvucci, A. Salzburger, D. Sammel, D. Sampsonidis, A. Sanchez, J. Sánchez, V. Sanchez Martinez, H. Sandaker, R. L. Sandbach, H. G. Sander, M. Sandhoff, C. Sandoval, D. P. C. Sankey, M. Sannino, A. Sansoni, C. Santoni, R. Santonico, H. Santos, I. Santoyo Castillo, K. Sapp, A. Sapronov, J. G. Saraiva, B. Sarrazin, O. Sasaki, K. Sato, E. Sauvan, G. Savage, P. Savard, N. Savic, C. Sawyer, L. Sawyer, J. Saxon, C. Sbarra, A. Sbrizzi, T. Scanlon, D. A. Scannicchio, M. Scarcella, V. Scarfone, J. Schaarschmidt, P. Schacht, B. M. Schachtner, D. Schaefer, L. Schaefer, R. Schaefer, J. Schaeffer, S. Schaepe, S. Schaetzel, U. Schäfer, A. C. Schaffer, D. Schaile, R. D. Schamberger, V. Scharf, V. A. Schegelsky, D. Scheirich, M. Schernau, C. Schiavi, S. Schier, C. Schillo, M. Schioppa, S. Schlenker, K. R. Schmidt-Sommerfeld, K. Schmieden, C. Schmitt, S. Schmitt, S. Schmitz, B. Schneider, U. Schnoor, L. Schoeffel, A. Schoening, B. D. Schoenrock, E. Schopf, M. Schott, J. F. P. Schouwenberg, J. Schovancova, S. Schramm, M. Schreyer, N. Schuh, A. Schulte, M. J. Schultens, H. -C. Schultz-Coulon, H. Schulz, M. Schumacher, B. A. Schumm, Ph. Schune, A. Schwartzman, T. A. Schwarz, H. Schweiger, Ph. Schwemling, R. Schwienhorst, J. Schwindling, T. Schwindt, G. Sciolla, F. Scuri, F. Scutti, J. Searcy, P. Seema, S. C. Seidel, A. Seiden, F. Seifert, J. M. Seixas, G. Sekhniaidze, K. Sekhon, S. J. Sekula, D. M. Seliverstov, N. Semprini-Cesari, C. Serfon, L. Serin, L. Serkin, M. Sessa, R. Seuster, H. Severini, T. Sfiligoj, F. Sforza, A. Sfyrla, E. Shabalina, N. W. Shaikh, L. Y. Shan, R. Shang, J. T. Shank, M. Shapiro, P. B. Shatalov, K. Shaw, S. M. Shaw, A. Shcherbakova, C. Y. Shehu, P. Sherwood, L. Shi, S. Shimizu, C. O. Shimmin, M. Shimojima, S. Shirabe, M. Shiyakova, A. Shmeleva, D. Shoaleh Saadi, M. J. Shochet, S. Shojaii, D. R. Shope, S. Shrestha, E. Shulga, M. A. Shupe, P. Sicho, A. M. Sickles, P. E. Sidebo, O. Sidiropoulou, D. Sidorov, A. Sidoti, F. Siegert, Dj. Sijacki, J. Silva, S. B. Silverstein, V. Simak, Lj. Simic, S. Simion, E. Simioni, B. Simmons, D. Simon, M. Simon, P. Sinervo, N. B. Sinev, M. Sioli, G. Siragusa, S. Yu. Sivoklokov, J. Sjölin, M. B. Skinner, H. P. Skottowe, P. Skubic, M. Slater, T. Slavicek, M. Slawinska, K. Sliwa, R. Slovak, V. Smakhtin, B. H. Smart, L. Smestad, J. Smiesko, S. Yu. Smirnov, Y. Smirnov, L. N. Smirnova, O. Smirnova, M. N. K. Smith, R. W. Smith, M. Smizanska, K. Smolek, A. A. Snesarev, I. M. Snyder, S. Snyder, R. Sobie, F. Socher, A. Soffer, D. A. Soh, G. Sokhrannyi, C. A. Solans Sanchez, M. Solar, E. Yu. Soldatov, U. Soldevila, A. A. Solodkov, A. Soloshenko, O. V. Solovyanov, V. Solovyev, P. Sommer, H. Son, H. Y. Song, A. Sood, A. Sopczak, V. Sopko, V. Sorin, D. Sosa, C. L. Sotiropoulou, R. Soualah, A. M. Soukharev, D. South, B. C. Sowden, S. Spagnolo, M. Spalla, M. Spangenberg, F. Spanò, D. Sperlich, F. Spettel, R. Spighi, G. Spigo, L. A. Spiller, M. Spousta, R. D. St. Denis, A. Stabile, R. Stamen, S. Stamm, E. Stanecka, R. W. Stanek, C. Stanescu, M. Stanescu-Bellu, M. M. Stanitzki, S. Stapnes, E. A. Starchenko, G. H. Stark, J. Stark, P. Staroba, P. Starovoitov, S. Stärz, R. Staszewski, P. Steinberg, B. Stelzer, H. J. Stelzer, O. Stelzer-Chilton, H. Stenzel, G. A. Stewart, J. A. Stillings, M. C. Stockton, M. Stoebe, G. Stoicea, P. Stolte, S. Stonjek, A. R. Stradling, A. Straessner, M. E. Stramaglia, J. Strandberg, S. Strandberg, A. Strandlie, M. Strauss, P. Strizenec, R. Ströhmer, D. M. Strom, R. Stroynowski, A. Strubig, S. A. Stucci, B. Stugu, N. A. Styles, D. Su, J. Su, S. Suchek, Y. Sugaya, M. Suk, V. V. Sulin, S. Sultansoy, T. Sumida, S. Sun, X. Sun, J. E. Sundermann, K. Suruliz, G. Susinno, M. R. Sutton, S. Suzuki, M. Svatos, M. Swiatlowski, I. Sykora, T. Sykora, D. Ta, C. Taccini, K. Tackmann, J. Taenzer, A. Taffard, R. Tafirout, N. Taiblum, H. Takai, R. Takashima, T. Takeshita, Y. Takubo, M. Talby, A. A. Talyshev, K. G. Tan, J. Tanaka, M. Tanaka, R. Tanaka, S. Tanaka, R. Tanioka, B. B. Tannenwald, S. Tapia Araya, S. Tapprogge, S. Tarem, G. F. Tartarelli, P. Tas, M. Tasevsky, T. Tashiro, E. Tassi, A. Tavares Delgado, Y. Tayalati, A. C. Taylor, G. N. Taylor, P. T. E. Taylor, W. Taylor, F. A. Teischinger, P. Teixeira-Dias, K. K. Temming, D. Temple, H. Ten Kate, P. K. Teng, J. J. Teoh, F. Tepel, S. Terada, K. Terashi, J. Terron, S. Terzo, M. Testa, R. J. Teuscher, T. Theveneaux-Pelzer, J. P. Thomas, J. Thomas-Wilsker, E. N. Thompson, P. D. Thompson, A. S. Thompson, L. A. Thomsen, E. Thomson, M. Thomson, M. J. Tibbetts, R. E. Ticse Torres, V. O. Tikhomirov, Yu. A. Tikhonov, S. Timoshenko, P. Tipton, S. Tisserant, K. Todome, T. Todorov, S. Todorova-Nova, J. Tojo, S. Tokár, K. Tokushuku, E. Tolley, L. Tomlinson, M. Tomoto, L. Tompkins, K. Toms, B. Tong, P. Tornambe, E. Torrence, H. Torres, E. Torró Pastor, J. Toth, F. Touchard, D. R. Tovey, T. Trefzger, A. Tricoli, I. M. Trigger, S. Trincaz-Duvoid, M. F. Tripiana, W. Trischuk, B. Trocmé, A. Trofymov, C. Troncon, M. Trottier-McDonald, M. Trovatelli, L. Truong, M. Trzebinski, A. Trzupek, J. C-L. Tseng, P. V. Tsiareshka, G. Tsipolitis, N. Tsirintanis, S. Tsiskaridze, V. Tsiskaridze, E. G. Tskhadadze, K. M. Tsui, I. I. Tsukerman, V. Tsulaia, S. Tsuno, D. Tsybychev, Y. Tu, A. Tudorache, V. Tudorache, A. N. Tuna, S. A. Tupputi, S. Turchikhin, D. Turecek, D. Turgeman, R. Turra, P. M. Tuts, M. Tyndel, G. Ucchielli, I. Ueda, M. Ughetto, F. Ukegawa, G. Unal, A. Undrus, G. Unel, F. C. Ungaro, Y. Unno, C. Unverdorben, J. Urban, P. Urquijo, P. Urrejola, G. Usai, L. Vacavant, V. Vacek, B. Vachon, C. Valderanis, E. Valdes Santurio, N. Valencic, S. Valentinetti, A. Valero, L. Valery, S. Valkar, J. A. Valls Ferrer, W. Van Den Wollenberg, P. C. Van Der Deijl, H. van der Graaf, N. van Eldik, P. van Gemmeren, J. Van Nieuwkoop, I. van Vulpen, M. C. van Woerden, M. Vanadia, W. Vandelli, R. Vanguri, A. Vaniachine, P. Vankov, G. Vardanyan, R. Vari, E. W. Varnes, T. Varol, D. Varouchas, A. Vartapetian, K. E. Varvell, J. G. Vasquez, G. A. Vasquez, F. Vazeille, T. Vazquez Schroeder, J. Veatch, V. Veeraraghavan, L. M. Veloce, F. Veloso, S. Veneziano, A. Ventura, M. Venturi, N. Venturi, A. Venturini, V. Vercesi, M. Verducci, W. Verkerke, J. C. Vermeulen, A. Vest, M. C. Vetterli, O. Viazlo, I. Vichou, T. Vickey, O. E. Vickey Boeriu, G. H. A. Viehhauser, S. Viel, L. Vigani, M. Villa, M. Villaplana Perez, E. Vilucchi, M. G. Vincter, V. B. Vinogradov, C. Vittori, I. Vivarelli, S. Vlachos, M. Vlasak, M. Vogel, P. Vokac, G. Volpi, M. Volpi, H. von der Schmitt, E. von Toerne, V. Vorobel, K. Vorobev, M. Vos, R. Voss, J. H. Vossebeld, N. Vranjes, M. Vranjes Milosavljevic, V. Vrba, M. Vreeswijk, R. Vuillermet, I. Vukotic, Z. Vykydal, P. Wagner, W. Wagner, H. Wahlberg, S. Wahrmund, J. Wakabayashi, J. Walder, R. Walker, W. Walkowiak, V. Wallangen, C. Wang, C. Wang, F. Wang, H. Wang, H. Wang, J. Wang, J. Wang, K. Wang, R. Wang, S. M. Wang, T. Wang, T. Wang, W. Wang, X. Wang, C. Wanotayaroj, A. Warburton, C. P. Ward, D. R. Wardrope, A. Washbrook, P. M. Watkins, A. T. Watson, M. F. Watson, G. Watts, S. Watts, B. M. Waugh, S. Webb, M. S. Weber, S. W. Weber, S. A. Weber, J. S. Webster, A. R. Weidberg, B. Weinert, J. Weingarten, C. Weiser, H. Weits, P. S. Wells, T. Wenaus, T. Wengler, S. Wenig, N. Wermes, M. Werner, M. D. Werner, P. Werner, M. Wessels, J. Wetter, K. Whalen, N. L. Whallon, A. M. Wharton, A. White, M. J. White, R. White, D. Whiteson, F. J. Wickens, W. Wiedenmann, M. Wielers, C. Wiglesworth, L. A. M. Wiik-Fuchs, A. Wildauer, F. Wilk, H. G. Wilkens, H. H. Williams, S. Williams, C. Willis, S. Willocq, J. A. Wilson, I. Wingerter-Seez, F. Winklmeier, O. J. Winston, B. T. Winter, M. Wittgen, J. Wittkowski, T. M. H. Wolf, M. W. Wolter, H. Wolters, S. D. Worm, B. K. Wosiek, J. Wotschack, M. J. Woudstra, K. W. Wozniak, M. Wu, M. Wu, S. L. Wu, X. Wu, Y. Wu, T. R. Wyatt, B. M. Wynne, S. Xella, D. Xu, L. Xu, B. Yabsley, S. Yacoob, D. Yamaguchi, Y. Yamaguchi, A. Yamamoto, S. Yamamoto, T. Yamanaka, K. Yamauchi, Y. Yamazaki, Z. Yan, H. Yang, H. Yang, Y. Yang, Z. Yang, W-M. Yao, Y. C. Yap, Y. Yasu, E. Yatsenko, K. H. Yau Wong, J. Ye, S. Ye, I. Yeletskikh, A. L. Yen, E. Yildirim, K. Yorita, R. Yoshida, K. Yoshihara, C. Young, C. J. S. Young, S. Youssef, D. R. Yu, J. Yu, J. M. Yu, J. Yu, L. Yuan, S. P. Y. Yuen, I. Yusuff, B. Zabinski, R. Zaidan, A. M. Zaitsev, N. Zakharchuk, J. Zalieckas, A. Zaman, S. Zambito, L. Zanello, D. Zanzi, A. G. Zecchinelli, C. Zeitnitz, M. Zeman, A. Zemla, J. C. Zeng, Q. Zeng, K. Zengel, O. Zenin, T. Ženiš, D. Zerwas, D. Zhang, F. Zhang, G. Zhang, H. Zhang, J. Zhang, L. Zhang, R. Zhang, R. Zhang, X. Zhang, Z. Zhang, X. Zhao, Y. Zhao, Z. Zhao, A. Zhemchugov, J. Zhong, B. Zhou, C. Zhou, L. Zhou, L. Zhou, M. Zhou, N. Zhou, C. G. Zhu, H. Zhu, J. Zhu, Y. Zhu, X. Zhuang, K. Zhukov, A. Zibell, D. Zieminska, N. I. Zimine, C. Zimmermann, S. Zimmermann, Z. Zinonos, M. Zinser, M. Ziolkowski, L. Živković, G. Zobernig, A. Zoccoli, M. zur Nedden, L. Zwalinski, ATLAS Collaboration

**Affiliations:** 1 Department of Physics, University of Adelaide, Adelaide, Australia; 2 Physics Department, SUNY Albany, Albany, NY USA; 3 Department of Physics, University of Alberta, Edmonton, AB Canada; 4 Department of Physics, Ankara University, Ankara, Turkey; 5 Istanbul Aydin University, Istanbul, Turkey; 6 Division of Physics, TOBB University of Economics and Technology, Ankara, Turkey; 7 LAPP, CNRS/IN2P3 and Université Savoie Mont Blanc, Annecy-le-Vieux, France; 8 High Energy Physics Division, Argonne National Laboratory, Argonne, IL USA; 9 Department of Physics, University of Arizona, Tucson, AZ USA; 10 Department of Physics, The University of Texas at Arlington, Arlington, TX USA; 11 Physics Department, University of Athens, Athens, Greece; 12 Physics Department, National Technical University of Athens, Zografou, Greece; 13 Department of Physics, The University of Texas at Austin, Austin, TX USA; 14 Institute of Physics, Azerbaijan Academy of Sciences, Baku, Azerbaijan; 15 Institut de Física d’Altes Energies (IFAE), The Barcelona Institute of Science and Technology, Barcelona, Spain; 16 Institute of Physics, University of Belgrade, Belgrade, Serbia; 17 Department for Physics and Technology, University of Bergen, Bergen, Norway; 18 Physics Division, Lawrence Berkeley National Laboratory and University of California, Berkeley, CA USA; 19 Department of Physics, Humboldt University, Berlin, Germany; 20 Albert Einstein Center for Fundamental Physics and Laboratory for High Energy Physics, University of Bern, Bern, Switzerland; 21 School of Physics and Astronomy, University of Birmingham, Birmingham, UK; 22 Department of Physics, Bogazici University, Istanbul, Turkey; 23 Department of Physics Engineering, Gaziantep University, Gaziantep, Turkey; 24 Faculty of Engineering and Natural Sciences, Istanbul Bilgi University, Istanbul, Turkey; 25 Faculty of Engineering and Natural Sciences, Bahcesehir University, Istanbul, Turkey; 26 Centro de Investigaciones, Universidad Antonio Narino, Bogotá, Colombia; 27 INFN Sezione di Bologna, Bologna, Italy; 28 Dipartimento di Fisica e Astronomia, Università di Bologna, Bologna, Italy; 29 Physikalisches Institut, University of Bonn, Bonn, Germany; 30 Department of Physics, Boston University, Boston, MA USA; 31 Department of Physics, Brandeis University, Waltham, MA USA; 32 Universidade Federal do Rio De Janeiro COPPE/EE/IF, Rio de Janeiro, Brazil; 33 Electrical Circuits Department, Federal University of Juiz de Fora (UFJF), Juiz de Fora, Brazil; 34 Federal University of Sao Joao del Rei (UFSJ), Sao Joao del Rei, Brazil; 35 Instituto de Fisica, Universidade de Sao Paulo, São Paulo, Brazil; 36 Physics Department, Brookhaven National Laboratory, Upton, NY USA; 37 Transilvania University of Brasov, Brasov, Romania; 38 National Institute of Physics and Nuclear Engineering, Bucharest, Romania; 39 Physics Department, National Institute for Research and Development of Isotopic and Molecular Technologies, Cluj Napoca, Romania; 40 University Politehnica Bucharest, Bucharest, Romania; 41 West University in Timisoara, Timisoara, Romania; 42 Departamento de Física, Universidad de Buenos Aires, Buenos Aires, Argentina; 43 Cavendish Laboratory, University of Cambridge, Cambridge, UK; 44 Department of Physics, Carleton University, Ottawa, ON Canada; 45 CERN, Geneva, Switzerland; 46 Enrico Fermi Institute, University of Chicago, Chicago, IL USA; 47 Departamento de Física, Pontificia Universidad Católica de Chile, Santiago, Chile; 48 Departamento de Física, Universidad Técnica Federico Santa María, Valparaiso, Chile; 49 Institute of High Energy Physics, Chinese Academy of Sciences, Beijing, China; 50 Department of Modern Physics, University of Science and Technology of China, Hefei, Anhui China; 51 Department of Physics, Nanjing University, Nanjing, Jiangsu China; 52 School of Physics, Shandong University, Jinan, Shandong China; 53 Shanghai Key Laboratory for Particle Physics and Cosmology, Department of Physics and Astronomy, Shanghai Jiao Tong University (also affiliated with PKU-CHEP), Shanghai, China; 54 Physics Department, Tsinghua University, Beijing, 100084 China; 55 Laboratoire de Physique Corpusculaire, Clermont Université and Université Blaise Pascal and CNRS/IN2P3, Clermont-Ferrand, France; 56 Nevis Laboratory, Columbia University, Irvington, NY USA; 57 Niels Bohr Institute, University of Copenhagen, Kobenhavn, Denmark; 58 INFN Gruppo Collegato di Cosenza, Laboratori Nazionali di Frascati, Frascati, Italy; 59 Dipartimento di Fisica, Università della Calabria, Rende, Italy; 60 Faculty of Physics and Applied Computer Science, AGH University of Science and Technology, Kraków, Poland; 61 Marian Smoluchowski Institute of Physics, Jagiellonian University, Kraków, Poland; 62 Institute of Nuclear Physics, Polish Academy of Sciences, Kraków, Poland; 63 Physics Department, Southern Methodist University, Dallas, TX USA; 64 Physics Department, University of Texas at Dallas, Richardson, TX USA; 65 DESY, Hamburg and Zeuthen, Hamburg, Germany; 66 Lehrstuh für Experimentelle Physik IV, Technische Universität Dortmund, Dortmund, Germany; 67 Institut für Kern- und Teilchenphysik, Technische Universität Dresden, Dresden, Germany; 68 Department of Physics, Duke University, Durham, NC USA; 69 SUPA-School of Physics and Astronomy, University of Edinburgh, Edinburgh, UK; 70 INFN Laboratori Nazionali di Frascati, Frascati, Italy; 71 Fakultät für Mathematik und Physik, Albert-Ludwigs-Universität, Freiburg, Germany; 72 Section de Physique, Université de Genève, Geneva, Switzerland; 73 INFN Sezione di Genova, Genoa, Italy; 74 Dipartimento di Fisica, Università di Genova, Genoa, Italy; 75 E. Andronikashvili Institute of Physics, Iv. Javakhishvili Tbilisi State University, Tbilisi, Georgia; 76 High Energy Physics Institute, Tbilisi State University, Tbilisi, Georgia; 77 II Physikalisches Institut, Justus-Liebig-Universität Giessen, Giessen, Germany; 78 SUPA-School of Physics and Astronomy, University of Glasgow, Glasgow, UK; 79 II Physikalisches Institut, Georg-August-Universität, Göttingen, Germany; 80 Laboratoire de Physique Subatomique et de Cosmologie, Université Grenoble-Alpes, CNRS/IN2P3, Grenoble, France; 81 Laboratory for Particle Physics and Cosmology, Harvard University, Cambridge, MA USA; 82 Kirchhoff-Institut für Physik, Ruprecht-Karls-Universität Heidelberg, Heidelberg, Germany; 83 Physikalisches Institut, Ruprecht-Karls-Universität Heidelberg, Heidelberg, Germany; 84 ZITI Institut für technische Informatik, Ruprecht-Karls-Universität Heidelberg, Mannheim, Germany; 85 Faculty of Applied Information Science, Hiroshima Institute of Technology, Hiroshima, Japan; 86 Department of Physics, The Chinese University of Hong Kong, Shatin, NT Hong Kong; 87 Department of Physics, The University of Hong Kong, Hong Kong, China; 88 Department of Physics, The Hong Kong University of Science and Technology, Clear Water Bay, Kowloon, Hong Kong, China; 89 Department of Physics, Indiana University, Bloomington, IN USA; 90 Institut für Astro- und Teilchenphysik, Leopold-Franzens-Universität, Innsbruck, Austria; 91 University of Iowa, Iowa City, IA USA; 92 Department of Physics and Astronomy, Iowa State University, Ames, IA USA; 93 Joint Institute for Nuclear Research, JINR Dubna, Dubna, Russia; 94 KEK, High Energy Accelerator Research Organization, Tsukuba, Japan; 95 Graduate School of Science, Kobe University, Kobe, Japan; 96 Faculty of Science, Kyoto University, Kyoto, Japan; 97 Kyoto University of Education, Kyoto, Japan; 98 Department of Physics, Kyushu University, Fukuoka, Japan; 99 Instituto de Física La Plata, Universidad Nacional de La Plata and CONICET, La Plata, Argentina; 100 Physics Department, Lancaster University, Lancaster, UK; 101 INFN Sezione di Lecce, Lecce, Italy; 102 Dipartimento di Matematica e Fisica, Università del Salento, Lecce, Italy; 103 Oliver Lodge Laboratory, University of Liverpool, Liverpool, UK; 104 Department of Physics, Jožef Stefan Institute and University of Ljubljana, Ljubljana, Slovenia; 105 School of Physics and Astronomy, Queen Mary University of London, London, UK; 106 Department of Physics, Royal Holloway University of London, Surrey, UK; 107 Department of Physics and Astronomy, University College London, London, UK; 108 Louisiana Tech University, Ruston, LA USA; 109 Laboratoire de Physique Nucléaire et de Hautes Energies, UPMC and Université Paris-Diderot and CNRS/IN2P3, Paris, France; 110 Fysiska institutionen, Lunds universitet, Lund, Sweden; 111 Departamento de Fisica Teorica C-15, Universidad Autonoma de Madrid, Madrid, Spain; 112 Institut für Physik, Universität Mainz, Mainz, Germany; 113 School of Physics and Astronomy, University of Manchester, Manchester, UK; 114 CPPM, Aix-Marseille Université and CNRS/IN2P3, Marseille, France; 115 Department of Physics, University of Massachusetts, Amherst, MA USA; 116 Department of Physics, McGill University, Montreal, QC Canada; 117 School of Physics, University of Melbourne, Melbourne, VIC Australia; 118 Department of Physics, The University of Michigan, Ann Arbor, MI USA; 119 Department of Physics and Astronomy, Michigan State University, East Lansing, MI USA; 120 INFN Sezione di Milano, Milan, Italy; 121 Dipartimento di Fisica, Università di Milano, Milan, Italy; 122 B.I. Stepanov Institute of Physics, National Academy of Sciences of Belarus, Minsk, Republic of Belarus; 123 National Scientific and Educational Centre for Particle and High Energy Physics, Minsk, Republic of Belarus; 124 Group of Particle Physics, University of Montreal, Montreal, QC Canada; 125 P.N. Lebedev Physical Institute of the Russian, Academy of Sciences, Moscow, Russia; 126 Institute for Theoretical and Experimental Physics (ITEP), Moscow, Russia; 127 National Research Nuclear University MEPhI, Moscow, Russia; 128 D.V. Skobeltsyn Institute of Nuclear Physics, M.V. Lomonosov Moscow State University, Moscow, Russia; 129 Fakultät für Physik, Ludwig-Maximilians-Universität München, Munich, Germany; 130 Max-Planck-Institut für Physik (Werner-Heisenberg-Institut), Munich, Germany; 131 Nagasaki Institute of Applied Science, Nagasaki, Japan; 132 Graduate School of Science and Kobayashi-Maskawa Institute, Nagoya University, Nagoya, Japan; 133 INFN Sezione di Napoli, Naples, Italy; 134 Dipartimento di Fisica, Università di Napoli, Naples, Italy; 135 Department of Physics and Astronomy, University of New Mexico, Albuquerque, NM USA; 136 Institute for Mathematics, Astrophysics and Particle Physics, Radboud University Nijmegen/Nikhef, Nijmegen, The Netherlands; 137 Nikhef National Institute for Subatomic Physics and University of Amsterdam, Amsterdam, The Netherlands; 138 Department of Physics, Northern Illinois University, DeKalb, IL USA; 139 Budker Institute of Nuclear Physics, SB RAS, Novosibirsk, Russia; 140 Department of Physics, New York University, New York, NY USA; 141 Ohio State University, Columbus, OH USA; 142 Faculty of Science, Okayama University, Okayama, Japan; 143 Homer L. Dodge Department of Physics and Astronomy, University of Oklahoma, Norman, OK USA; 144 Department of Physics, Oklahoma State University, Stillwater, OK USA; 145 Palacký University, RCPTM, Olomouc, Czech Republic; 146 Center for High Energy Physics, University of Oregon, Eugene, OR USA; 147 LAL, Univ. Paris-Sud, CNRS/IN2P3, Université Paris-Saclay, Orsay, France; 148 Graduate School of Science, Osaka University, Osaka, Japan; 149 Department of Physics, University of Oslo, Oslo, Norway; 150 Department of Physics, Oxford University, Oxford, UK; 151 INFN Sezione di Pavia, Pavia, Italy; 152 Dipartimento di Fisica, Università di Pavia, Pavia, Italy; 153 Department of Physics, University of Pennsylvania, Philadelphia, PA USA; 154 National Research Centre “Kurchatov Institute” B.P. Konstantinov Petersburg Nuclear Physics Institute, St. Petersburg, Russia; 155 INFN Sezione di Pisa, Pisa, Italy; 156 Dipartimento di Fisica E. Fermi, Università di Pisa, Pisa, Italy; 157 Department of Physics and Astronomy, University of Pittsburgh, Pittsburgh, PA USA; 158 Laboratório de Instrumentação e Física Experimental de Partículas-LIP, Lisbon, Portugal; 159 Faculdade de Ciências, Universidade de Lisboa, Lisbon, Portugal; 160 Department of Physics, University of Coimbra, Coimbra, Portugal; 161 Centro de Física Nuclear da Universidade de Lisboa, Lisbon, Portugal; 162 Departamento de Fisica, Universidade do Minho, Braga, Portugal; 163 Departamento de Fisica Teorica y del Cosmos and CAFPE, Universidad de Granada, Granada, Spain; 164 Dep Fisica and CEFITEC of Faculdade de Ciencias e Tecnologia, Universidade Nova de Lisboa, Caparica, Portugal; 165 Institute of Physics, Academy of Sciences of the Czech Republic, Prague, Czech Republic; 166 Czech Technical University in Prague, Prague, Czech Republic; 167 Faculty of Mathematics and Physics, Charles University in Prague, Prague, Czech Republic; 168 State Research Center Institute for High Energy Physics (Protvino), NRC KI, Protvino, Russia; 169 Particle Physics Department, Rutherford Appleton Laboratory, Didcot, UK; 170 INFN Sezione di Roma, Rome, Italy; 171 Dipartimento di Fisica, Sapienza Università di Roma, Rome, Italy; 172 INFN Sezione di Roma Tor Vergata, Rome, Italy; 173 Dipartimento di Fisica, Università di Roma Tor Vergata, Rome, Italy; 174 INFN Sezione di Roma Tre, Rome, Italy; 175 Dipartimento di Matematica e Fisica, Università Roma Tre, Rome, Italy; 176 Faculté des Sciences Ain Chock, Réseau Universitaire de Physique des Hautes Energies-Université Hassan II, Casablanca, Morocco; 177 Centre National de l’Energie des Sciences Techniques Nucleaires, Rabat, Morocco; 178 Faculté des Sciences Semlalia, Université Cadi Ayyad, LPHEA-Marrakech, Marrakech, Morocco; 179 Faculté des Sciences, Université Mohamed Premier and LPTPM, Oujda, Morocco; 180 Faculté des Sciences, Université Mohammed V, Rabat, Morocco; 181 DSM/IRFU (Institut de Recherches sur les Lois Fondamentales de l’Univers), CEA Saclay (Commissariat à l’Energie Atomique et aux Energies Alternatives), Gif-sur-Yvette, France; 182 Santa Cruz Institute for Particle Physics, University of California Santa Cruz, Santa Cruz, CA USA; 183 Department of Physics, University of Washington, Seattle, WA USA; 184 Department of Physics and Astronomy, University of Sheffield, Sheffield, UK; 185 Department of Physics, Shinshu University, Nagano, Japan; 186 Fachbereich Physik, Universität Siegen, Siegen, Germany; 187 Department of Physics, Simon Fraser University, Burnaby, BC Canada; 188 SLAC National Accelerator Laboratory, Stanford, CA USA; 189 Faculty of Mathematics, Physics and Informatics, Comenius University, Bratislava, Slovak Republic; 190 Department of Subnuclear Physics, Institute of Experimental Physics of the Slovak Academy of Sciences, Kosice, Slovak Republic; 191 Department of Physics, University of Cape Town, Cape Town, South Africa; 192 Department of Physics, University of Johannesburg, Johannesburg, South Africa; 193 School of Physics, University of the Witwatersrand, Johannesburg, South Africa; 194 Department of Physics, Stockholm University, Stockholm, Sweden; 195 The Oskar Klein Centre, Stockholm, Sweden; 196 Physics Department, Royal Institute of Technology, Stockholm, Sweden; 197 Departments of Physics and Astronomy and Chemistry, Stony Brook University, Stony Brook, NY USA; 198 Department of Physics and Astronomy, University of Sussex, Brighton, UK; 199 School of Physics, University of Sydney, Sydney, Australia; 200 Institute of Physics, Academia Sinica, Taipei, Taiwan; 201 Department of Physics, Technion: Israel Institute of Technology, Haifa, Israel; 202 Raymond and Beverly Sackler School of Physics and Astronomy, Tel Aviv University, Tel Aviv, Israel; 203 Department of Physics, Aristotle University of Thessaloniki, Thessaloníki, Greece; 204 International Center for Elementary Particle Physics and Department of Physics, The University of Tokyo, Tokyo, Japan; 205 Graduate School of Science and Technology, Tokyo Metropolitan University, Tokyo, Japan; 206 Department of Physics, Tokyo Institute of Technology, Tokyo, Japan; 207 Department of Physics, University of Toronto, Toronto, ON Canada; 208 TRIUMF, Vancouver, BC Canada; 209 Department of Physics and Astronomy, York University, Toronto, ON Canada; 210 Faculty of Pure and Applied Sciences, and Center for Integrated Research in Fundamental Science and Engineering, University of Tsukuba, Tsukuba, Japan; 211 Department of Physics and Astronomy, Tufts University, Medford, MA USA; 212 Department of Physics and Astronomy, University of California Irvine, Irvine, CA USA; 213 INFN Gruppo Collegato di Udine, Sezione di Trieste, Udine, Italy; 214 ICTP, Trieste, Italy; 215 Dipartimento di Chimica Fisica e Ambiente, Università di Udine, Udine, Italy; 216 Department of Physics and Astronomy, University of Uppsala, Uppsala, Sweden; 217 Department of Physics, University of Illinois, Urbana, IL USA; 218 Instituto de Fisica Corpuscular (IFIC) and Departamento de Fisica Atomica, Molecular y Nuclear and Departamento de Ingeniería Electrónica and Instituto de Microelectrónica de Barcelona (IMB-CNM), University of Valencia and CSIC, Valencia, Spain; 219 Department of Physics, University of British Columbia, Vancouver, BC Canada; 220 Department of Physics and Astronomy, University of Victoria, Victoria, BC Canada; 221 Department of Physics, University of Warwick, Coventry, UK; 222 Waseda University, Tokyo, Japan; 223 Department of Particle Physics, The Weizmann Institute of Science, Rehovot, Israel; 224 Department of Physics, University of Wisconsin, Madison, WI USA; 225 Fakultät für Physik und Astronomie, Julius-Maximilians-Universität, Würzburg, Germany; 226 Fakultät für Mathematik und Naturwissenschaften, Fachgruppe Physik, Bergische Universität Wuppertal, Wuppertal, Germany; 227 Department of Physics, Yale University, New Haven, CT USA; 228 Yerevan Physics Institute, Yerevan, Armenia; 229 Centre de Calcul de l’Institut National de Physique Nucléaire et de Physique des Particules (IN2P3), Villeurbanne, France; 230 CERN, 1211 Geneva 23, Switzerland

## Abstract

A search has been made for supersymmetry in a final state containing two photons and missing transverse momentum using the ATLAS detector at the Large Hadron Collider. The search makes use of $3.2{~\mathrm{fb}^{-1}}$ of proton-proton collision data collected at a centre-of-mass energy of 13 TeV in 2015. Using a combination of data-driven and Monte-Carlo-based approaches, the Standard Model background is estimated to be $0.27^{+0.22}_{-0.10}$ events. No events are observed in the signal region; considering the expected background and its uncertainty, this observation implies a model-independent 95 % CL upper limit of 0.93 fb (3.0 events) on the visible cross section due to physics beyond the Standard Model. In the context of a generalized model of gauge-mediated supersymmetry breaking with a bino-like next-to-lightest supersymmetric particle, this leads to a lower limit of 1650 GeV on the mass of a degenerate octet of gluino states, independent of the mass of the lighter bino-like neutralino.

## Introduction

This paper presents a search for signatures of supersymmetry in events containing two energetic isolated photons and large missing transverse momentum (with magnitude denoted $E_{\text {T}}^{\text {miss}}$) in $3.2{~\mathrm{fb}^{-1}}$ of proton–proton (*pp*) collision data at $\sqrt{s}=13$ TeV recorded with the ATLAS detector at the Large Hadron Collider (LHC) in 2015. The results are interpreted in the context of general gauge mediation (GGM) [1, 2] models that include the production of supersymmetric partners of Standard Model (SM) particles that possess color charge. In all models of GGM, the lightest supersymmetric particle (LSP) is the gravitino $\tilde{G}$ (the partner of the hypothetical quantum of the gravitational field), with a mass significantly less than 1 GeV. In the GGM model considered here, the decay of the supersymmetric states produced in *pp* collisions would proceed through the next-to-lightest supersymmetric particle (NLSP), which would then decay to the $\tilde{G}$ LSP and one or more SM particles, with a high probability of decay into $\gamma$ + $\tilde{G}$. All accessible supersymmetric states with the exception of the $\tilde{G}$ are assumed to be short-lived, leading to prompt production of SM particles that would be observed in the ATLAS detector. These results extend those of prior studies with 8 TeV collision data from Run 1 by the ATLAS [3] and CMS [4] experiments.

Supersymmetry (SUSY) [5–10] introduces a symmetry between fermions and bosons, resulting in a SUSY particle (sparticle) with identical quantum numbers, with the exception of a difference of half a unit of spin relative to its corresponding SM partner. If SUSY were an exact symmetry of nature, each sparticle would have a mass equal to that of its SM partner. Since no sparticles have yet been observed, SUSY would have to be a broken symmetry. Assuming *R*-parity conservation [11], sparticles are produced in pairs. These would then decay through cascades involving other sparticles until the stable, undetectable LSP is produced, leading to a final state with significant $E_{\text {T}}^{\text {miss}}$.

Experimental signatures of gauge-mediated supersymmetry-breaking models [12–14] are largely determined by the nature of the NLSP. For GGM, the NLSP is often formed from an admixture of any of the SUSY partners of the electroweak gauge and Higgs boson states. In this study the NLSP, assumed to be electrically neutral and purely bino-like (the SUSY partner of the SM U(1) gauge boson), is the lightest gaugino state $\tilde{\chi }^{0}_{1}$. In this case, the final decay in each of the two cascades in a GGM event would be predominantly $\tilde{\chi }^{0}_{1}\rightarrow \gamma +\tilde{G}$, leading to final states with $\gamma \gamma +E_{\text {T}}^{\text {miss}}$.

**Fig. 1:**
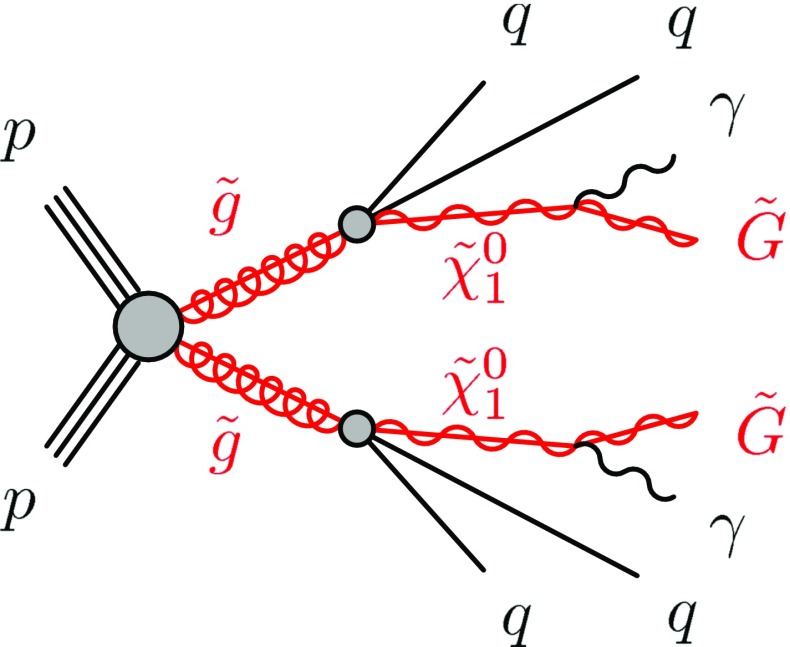
Typical production and decay-chain processes for the gluino-pair production GGM model for which the NLSP is a bino-like neutralino

In addition to the bino-like $\tilde{\chi }^{0}_{1}$ NLSP, a degenerate octet of gluinos (the SUSY partner of the SM gluon) is taken to be potentially accessible with 13 TeV *pp* collisions. Both the gluino and $\tilde{\chi }^{0}_{1}$ masses are considered to be free parameters, with the $\tilde{\chi }^{0}_{1}$ mass constrained to be less than that of the gluino. All other SUSY masses are set to values that preclude their production in 13 TeV *pp* collisions. This results in a SUSY production process that proceeds through the creation of pairs of gluino states, each of which subsequently decays via a virtual squark (the 12 squark flavour/chirality eigenstates are taken to be fully degenerate) to a quark–antiquark pair plus the NLSP neutralino. Additional SM objects (jets, leptons, photons) may be produced in these cascades. The $\tilde{\chi }^{0}_{1}$ branching fraction to $\gamma$ + $\tilde{G}$ is 100 % for $m_{\tilde{\chi }^{0}_{1}} \rightarrow 0$ and approaches $\cos ^2 \theta _W$ for $m_{\tilde{\chi }^{0}_{1}} \gg m_Z$, with the remainder of the $\tilde{\chi }^{0}_{1}$ sample decaying to *Z* + $\tilde{G}$. For all $\tilde{\chi }^{0}_{1}$ masses, then, the branching fraction is dominated by the photonic decay, leading to the diphoton-plus-$E_{\text {T}}^{\text {miss}}$signature. For this model with a bino-like NLSP, a typical production and decay channel for strong (gluino) production is exhibited in Fig. 1. Finally, it should be noted that the phenomenology relevant to this search has a negligible dependence on the ratio $\tan \beta$ of the two SUSY Higgs-doublet vacuum expectation values; for this analysis $\tan \beta$ is set to 1.5.

## Samples of simulated processes

For the GGM models under study, the SUSY mass spectra and branching fractions are calculated using SUSPECT 2.41 [15] and SDECAY 1.3b [16], respectively, inside the package SUSY-HIT 1.3 [17]. The Monte Carlo (MC) SUSY signal samples are produced using Herwig++ 2.7.1 [18] with CTEQ6L1 parton distribution functions (PDFs) [19]. Signal cross sections are calculated to next-to-leading order (NLO) in the strong coupling constant, including, for the case of strong production, the resummation of soft gluon emission at next-to-leading-logarithmic accuracy (NLO+NLL) [20–24]. The nominal cross section and its uncertainty are taken from an envelope of cross-section predictions using different PDF sets and factorization and renormalization scales [25]. At fixed centre-of-mass energy, SUSY production cross sections decrease rapidly with increasing SUSY particle mass. At $\sqrt{s} = 13$ TeV, the gluino-pair production cross section is approximately 25 fb for a gluino mass of 1.4 TeV and falls to below 1 fb for a gluino mass of 2.0 TeV.

While most of the backgrounds to the GGM models under examination are estimated through the use of control samples selected from data, as described below, the extrapolation from control regions (CRs) to the signal region (SR) depends on simulated samples, as do the optimization studies. Diphoton, photon+jet, $W\gamma$, $Z\gamma$, $W\gamma \gamma$ and $Z\gamma \gamma$ SM processes are generated using the SHERPA 2.1.1 simulation package [26], making use of the CT10 PDFs [27]. The matrix elements are calculated with up to three parton emissions at leading order (four in the case of photon+jet samples) and merged with the SHERPA parton shower [28] using the ME+PS@LO prescription [29]. The $t\bar{t}\gamma$ process is generated using MadGraph5_aMC@NLO [30] with the CTEQ6L1 PDFs [19], in conjunction with PYTHIA 8.186 [31] with the NNPDF2.3LO PDF set [32, 33] and the A14 set [34] of tuned parameters.

All simulated samples are processed with a full ATLAS detector simulation [35] based on GEANT4 [36]. The effect of additional *pp* interactions per bunch crossing (“pile-up”) as a function of the instantaneous luminosity is taken into account by overlaying simulated minimum-bias events according to the observed distribution of the number of pile-up interactions in data, with an average of 13 interactions per event.

## ATLAS detector

The ATLAS experiment records *pp* collision data with a multipurpose detector [37] that has a forward-backward symmetric cylindrical geometry and nearly 4$\pi$ solid angle coverage. Closest to the beam line are solid-state tracking devices comprising layers of silicon-based pixel and strip detectors covering $\left| \eta \right| <2.5$ and straw-tube detectors covering $\left| \eta \right| <2.0$, located inside a thin superconducting solenoid that provides a 2T magnetic field. Outside of this “inner detector”, fine-grained lead/liquid-argon electromagnetic (EM) calorimeters provide coverage over $\left| \eta \right| < 3.2$ for the measurement of the energy and direction of electrons and photons. A presampler, covering $\left| \eta \right| < 1.8$, is used to correct for energy lost upstream of the EM calorimeter. A steel/scintillator-tile hadronic calorimeter covers the region $|\eta | < 1.7$, while a copper/liquid-argon medium is used for hadronic calorimeters in the end cap region $1.5< |\eta | < 3.2$. In the forward region $3.2< |\eta | < 4.9$ liquid-argon calorimeters with copper and tungsten absorbers measure the electromagnetic and hadronic energy. A muon spectrometer consisting of three superconducting toroidal magnet systems, each comprising eight toroidal coils, tracking chambers, and detectors for triggering, surrounds the calorimeter system. The muon system reconstructs penetrating tracks over a range $|\eta | < 2.7$ and provides input to the trigger system over a range $|\eta | < 2.4$. A two-level trigger system [38] is used to select events. The first-level trigger is implemented in hardware and uses a subset of the detector information to reduce the accepted rate to less than 100 kHz. This is followed by a software-based ’high-level’ trigger (HLT) that reduces the recorded event rate to approximately 1 kHz.

## Event reconstruction

Primary vertices are formed from sets of two or more tracks, each with transverse momentum $p_\mathrm{T}^{\mathrm {track}}>$ 400 MeV, that are mutually consistent with having originated at the same three-dimensional point within the luminous region of the colliding proton beams. When more than one such primary vertex is found, the vertex with the largest sum of the squared transverse momenta of the associated tracks is chosen.

Electron candidates are reconstructed from EM calorimeter energy clusters consistent in transverse shape and longitudinal development with having arisen from the impact of an electromagnetic particle (electron or photon) upon the face of the calorimeter. For the object to be considered an electron, it is required to match a track identified by a reconstruction algorithm optimized for recognizing charged particles with a high probability of bremsstrahlung [39]. The energy of the electron candidate is determined from the EM cluster, while its direction is determined from the associated reconstructed track. Electron candidates are required to have $p_{\text {T}}> 25~\text{GeV}$ and $|\eta | < 2.37$, and to be outside the transition region $1.37< \left| \eta \right| < 1.52$ between the central and forward portions of the EM calorimeter. Finally, the electron track is required to be consistent with originating from the primary vertex in both the $r-z$ and $r-\phi$ planes. Further details of the reconstruction of electrons can be found in Refs. [40] and [41].

Electromagnetic clusters are classified as photon candidates provided that they either have no matched track or have one or more matched tracks consistent with having arisen from a photon conversion. Based on the characteristics of the longitudinal and transverse shower development in the EM calorimeter, photons are classified as “loose” or “tight”, with the tight requirements leading to a more pure but less efficienct selection of photons relative to that of the loose requirements [42]. Photon candidates are required to have $p_{\text {T}}> 25~\text{GeV}$, to be within $\left| \eta \right| < 2.37$, and to be outside the transition region $1.37< \left| \eta \right| < 1.52$. Additionally, an isolation requirement is imposed: after correcting for contributions from pile-up and the deposition ascribed to the photon itself, the energy within a cone of $\Delta R = 0.4$ around the cluster barycentre is required to be less than $2.45~\text{GeV} + 0.022 \times p_{\mathrm {T}}^{\gamma }$, where $p_{\mathrm {T}}^{\gamma }$ is the transverse momentum of the cluster. In the case that an EM calorimeter deposition identified as a photon overlaps the cluster of an identified electron within a cone of $\Delta R = 0.4$, the photon candidate is discarded and the electron candidate is retained. Further details of the reconstruction of photons can be found in Ref. [42].

Muon candidates make use of reconstructed tracks from the inner detector as well as information from the muon system [43]. Muons are required to be either “combined”, for which the muon is reconstructed independently in both the muon spectrometer and the inner detector and then combined, or “segment-tagged”, for which the muon spectrometer is used to tag tracks as muons, without requiring a fully reconstructed candidate in the muon spectrometer. Muons are required to have $p_{\text {T}}> 25~\text{GeV}$ and $|\eta |<2.7$, with the muon track required to be consistent with originating from the primary vertex in both the $r-z$ and $r-\phi$ planes.

Jets are reconstructed from three-dimensional energy clusters [44] in the electromagnetic and hadronic calorimeters using the anti-$k_t$ algorithm [45] with a radius parameter *R* = 0.4. Each cluster is calibrated to the electromagnetic scale prior to jet reconstruction. The reconstructed jets are then calibrated to particle level by the application of a jet energy scale derived from simulation and *in situ* corrections based on 8 TeV data [46, 47]. In addition, the expected average energy contribution from pile-up clusters is subtracted using a factor dependent on the jet area [46]. Track-based selection requirements are applied to reject jets with $p_{\text {T}}{} < 60$ GeV and $|\eta | < 2.4$ that originate from pile-up interactions [48]. Once calibrated, jets are required to have $p_{\text {T}}>$ 40 GeV and $|\eta | < 2.8$.

To resolve the ambiguity that arises when a photon is also reconstructed as a jet, if a jet and a photon are reconstructed within an angular distance $\Delta R = 0.4$ of one another, the photon is retained and the jet is discarded. If a jet and an electron are reconstructed within an angular distance $\Delta R = 0.2$ of one another, the electron is retained and the jet is discarded; if $0.2< \Delta R < 0.4$ then the jet is retained and the electron is discarded. Finally, in order to suppress the reconstruction of muons arising from showers induced by jets, if a jet and a muon are found with $\Delta R < 0.4$ the jet is retained and the muon is discarded.

The missing transverse momentum $\varvec{p}_{\mathrm {T}}^\mathrm{miss}$ is defined as the negative vector sum of the $p_{\text {T}}$ of all reconstructed physics objects in the event, with an extra term added to account for soft energy in the event that is not associated with any of the objects. This “$E_{\text {T}}^{\text {miss}}$ soft term” is calculated from inner-detector tracks with $p_{\text {T}}$above 400 MeV matched to the primary vertex to make it less dependent upon pile-up contamination [49, 50]. The scalar observable $E_{\text {T}}^{\text {miss}}$ is defined to be the magnitude of the resulting $\varvec{p}_{\mathrm {T}}^\mathrm{miss}$ vector.

Several additional observables are defined to help in the discrimination of SM backgrounds from potential GGM signals. The total visible transverse energy, $H_{\text {T}}$, is calculated as the scalar sum of the transverse momenta of the reconstructed photons and any additional leptons and jets in the event. The “effective mass”, $m_{\mathrm {eff}}$, is defined as the scalar sum of $H_{\text {T}}$ and $E_{\text {T}}^{\text {miss}}$. The minimum jet–$\varvec{p}_{\mathrm {T}}^\mathrm{miss}$ separation, $\Delta \phi _{\mathrm {min}}(\mathrm {jet},\varvec{p}_{\mathrm {T}}^\mathrm{miss}{})$, is defined as the minimum azimuthal angle between the missing transverse momentum vector and the two leading (highest-$p_{\text {T}}$) jets with $p_{\text {T}}> 75$ GeV in the event, if they are present. If no such jets exist, no requirement is placed on this observable.

## Event selection

The data sample is selected by a HLT trigger requiring the presence of two loose photons, each with $p_{\text {T}}$ greater than 50 GeV. Offline, two tight photons with $p_{\text {T}}> 75~\text{GeV}$ are required. In order to ensure that $E_{\text {T}}^{\text {miss}}$ is measured well, events are removed from the data sample if they contain jets likely to be produced by beam backgrounds, cosmic rays or detector noise [51].

**Fig. 2:**
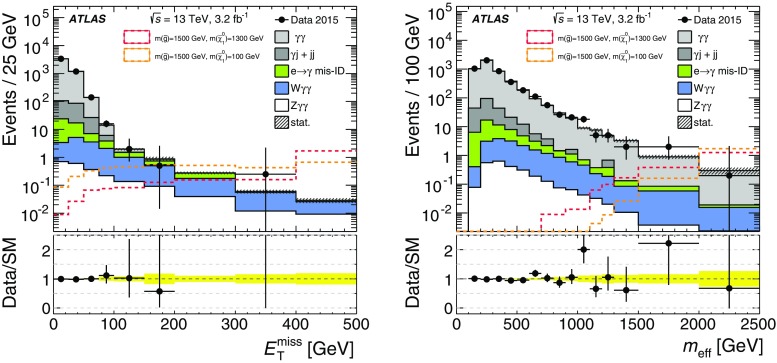
Distributions of $E_{\text {T}}^{\text {miss}}$(*left*) and $m_{\mathrm {eff}}$(*right*) for the diphoton sample after the application of requirements of $p_{\mathrm {T}}^{\gamma }> 75$ GeV on each selected photon and of $\Delta \phi _{\mathrm {min}}(\mathrm {jet},\varvec{p}_{\mathrm {T}}^\mathrm{miss}{})> 0.5$, but with no requirements imposed on $E_{\text {T}}^{\text {miss}}$ and $m_{\mathrm {eff}}$. The expected contributions from SM processes are estimated using the combination of Monte Carlo and data-driven estimates discussed in Sect. 6. Uncertainties (shaded bands for MC simulation, *error bars* for data) are statistical only. The *yellow band* represents the uncertainty in the data/SM ratio that arises from the statisical limitations of the estimates of the various expected sources of SM background. Also shown are the expected contributions from the GGM signal for the two benchmark points, $(m_{\tilde{g}},m_{\tilde{\chi }^{0}_{1}}) = (1500,1300)$ GeV and $(m_{\tilde{g}},m_{\tilde{\chi }^{0}_{1}}) = (1500,100)$ GeV. The final bin of each plot includes the ‘overflow’ contribution that lies above the nominal upper range of the plot

To exploit the significant undetectable transverse momentum carried away by the gravitinos, a requirement on $E_{\text {T}}^{\text {miss}}$ is imposed on the diphoton event sample. To take advantage of the high production energy scale associated with signal events near the expected reach of the analysis, an additional requirement on $m_{\mathrm {eff}}$ is applied. To further ensure the accurate reconstruction of $E_{\text {T}}^{\text {miss}}$ and to suppress backgrounds associated with the mismeasurement of hadronic jets, a requirement of $\Delta \phi _{\mathrm {min}}(\mathrm {jet},\varvec{p}_{\mathrm {T}}^\mathrm{miss}{})> 0.5$ is imposed. Figure 2 shows the $E_{\text {T}}^{\text {miss}}$ and $m_{\mathrm {eff}}$ distributions of the diphoton sample after the application of requirements of $p_{\mathrm {T}}^{\gamma }> 75$ GeV on each selected photon and of $\Delta \phi _{\mathrm {min}}(\mathrm {jet},\varvec{p}_{\mathrm {T}}^\mathrm{miss}{})> 0.5$, but with no requirements yet imposed on $E_{\text {T}}^{\text {miss}}$ and $m_{\mathrm {eff}}$. Also shown are the expected contributions from SM processes, estimated using the combination of Monte Carlo and data-driven estimates discussed in Sect. 6.

As discussed in Sect. 1, the GGM signal space is parameterized by the masses of the gluino ($m_{\tilde{g}}$) and bino-like NLSP ($m_{\tilde{\chi }^{0}_{1}}$). The sensitivity of this analysis was optimized for two signal scenarios near the expected reach in $m_{\tilde{g}}$: high and low neutralino-mass benchmark points were chosen with $(m_{\tilde{g}},m_{\tilde{\chi }^{0}_{1}}) = (1500,1300)$ GeV and $(m_{\tilde{g}},m_{\tilde{\chi }^{0}_{1}}) = (1500,100)$ GeV, respectively.

Based on background estimates derived from the MC samples described in Sect. 2, the selection requirements were optimized as a function of $E_{\text {T}}^{\text {miss}}$, $m_{\mathrm {eff}}$ and $p_{\mathrm {T}}^{\gamma }$ by maximizing the expected discovery sensitivity of the analysis, for each of the two signal benchmark points. The selected values of the minimum requirements on all three optimization parameters were found to be very similar for the low and high neutralino-mass benchmark points, leading to the definition of a single signal region (SR). The selection requirements for this SR are shown in Table 1.

**Table 1:** Requirements defining the signal region (SR) and the $W\gamma \gamma$ CR referred to in Sect. 6

SR	$W\gamma \gamma$ CR
2 Tight photons with $p_{\text {T}}> 75$ GeV	2 Tight photons with $p_{\text {T}}> 50$ GeV
	1 *e* or $\mu$ with $p_{\text {T}}> 25$ GeV
$\Delta \phi _{\mathrm {min}}(\mathrm {jet},\varvec{p}_{\mathrm {T}}^\mathrm{miss}{})> 0.5$	$\Delta \phi _{\mathrm {min}}(\mathrm {jet},\varvec{p}_{\mathrm {T}}^\mathrm{miss}{})> 0.5$
$E_{\text {T}}^{\text {miss}}> 175$ GeV	$50< E_{\text {T}}^{\text {miss}}< 175$ GeV
$m_{\mathrm {eff}}> 1500$ GeV	$N(\mathrm {jets}) < 3$
	$m_{e \gamma } \notin$ 83–97 GeV

## Background estimation

Processes that contribute to the Standard Model background of diphoton final states can be divided into three primary components. The largest contribution to the inclusive diphoton spectrum is the “QCD background”, which can be further divided into a contribution from two real photons produced in association with jets, and a “jet-faking-photon” contribution arising from $\gamma$+jet and multijet events for which one or both reconstructed photons are faked by a jet, typically by producing a $\pi ^{0}\rightarrow \gamma \gamma$ decay that is misidentified as a prompt photon. An “electron-faking-photon background” arises predominantly from *W*, *Z*, and $\; t\bar{t}$ events, possibly accompanied by additional jets and/or photons, for which an electron is misidentified as a photon. Electron-to-photon misidentification is due primarily to instances for which an electron radiates a high-momentum photon as it traverses the material of the ATLAS inner detector. Last, an “irreducible background” arises from $W\gamma \gamma$ and $Z\gamma \gamma$ events. These backgrounds are estimated with a combination of data-driven and simulation-based methods described as follows.

The component of the QCD background arising from real diphoton events ($\gamma \gamma$) is estimated directly from diphoton MC events, rescaled as function of $E_{\text {T}}^{\text {miss}}$and the number of selected jets to match the respective distributions for the inclusive diphoton sample in the range $E_{\text {T}}^{\text {miss}}<$ 100 GeV. While this background dominates the inclusive diphoton sample, it is very steeply falling in $E_{\text {T}}^{\text {miss}}$, making it small relative to backgrounds with real $E_{\text {T}}^{\text {miss}}$ for $E_{\text {T}}^{\text {miss}}\gtrsim$ 100 GeV, independent of the reweighting.

The component of the QCD background arising from jets faking photons and the background arising from electrons faking photons are both estimated with a data-driven “fake-factor” method, for which events in data samples enriched in the background of interest are weighted by factors parameterizing the misidentification rate.

To estimate the jet-faking-photon fake-factor, the jet-faking-photon background is enriched by using an inverted isolation requirement, selecting events only if they contain one or more non-isolated photons. The relative probability of an energy cluster being reconstructed as an isolated, rather than non-isolated, photon is known as the photon-isolation fake factor, and is measured in an orthogonal “non-tight” sample of photons. The selection of this sample requires that all the tight photon identification requirements be satisfied, with the exception that at least one of the requirements on the calorimeter variables defined only with the first (strip) layer of the electromagnetic calorimeter fails. This leads to a sample enriched in identified (non-tight) photons that are actually $\pi ^0$s within jets. The correlation between the isolation variable and the photon identification requirements was found to be small and to have no significant impact on the estimation of the jet-faking-photon fake-factor. The fake factors depend upon $p_{\text {T}}$ and $\eta$, and vary between 10 and 30 %. The jet-faking photon background is then estimated by weighting events with non-isolated photons by the applicable photon-isolation fake factor.

The electron-faking-photon background is estimated with a similar fake-factor method. For this case, the electron-faking-photon background is enriched by selecting events with a reconstructed electron instead of a second photon. Fake factors for electrons being misidentified as photons are then measured by comparing the ratio of reconstructed $e\gamma$ to *ee* events arising from *Z* bosons decaying to electron–positron pairs, selected within the mass range of 75–105 GeV. The electron-faking-photon background is then estimated by weighting selected $e\gamma$ events by their corresponding fake factors, which are typically a few percent.

The irreducible background from $W\gamma \gamma$ events is estimated with MC simulation; however, because it is a potentially dominant background contribution, the overall normalization is derived in a $\ell \gamma \gamma$ control region ($W\gamma \gamma$ CR) as follows. Events in the $W\gamma \gamma$ CR are required to have two tight, isolated photons with $p_{\text {T}}> 50$ GeV, and exactly one selected lepton (electron or muon) with $p_{\text {T}}> 25$ GeV. As with the SR, events are required to have $\Delta \phi _{\mathrm {min}}(\mathrm {jet},\varvec{p}_{\mathrm {T}}^\mathrm{miss}{})> 0.5$, so that the direction of the missing transverse momentum vector is not aligned with that of any high-$p_{\text {T}}$ jet. To ensure that the control sample has no overlap with the signal region, events are discarded if $E_{\text {T}}^{\text {miss}}> 175$ GeV. While these requirements target $W\gamma \gamma$ production, they also are expected to select appreciable backgrounds from $t\bar{t}\gamma$, $Z\gamma$ and $Z\gamma \gamma$ events, and thus additional requirements are applied. To suppress $t\bar{t}\gamma$ contributions to the $W\gamma \gamma$ CR, events are discarded if they contain more than two selected jets. To suppress $Z\gamma$ contributions, events are discarded if there is an *e*–$\gamma$ pair in the events with $83< m_{e\gamma } < 97$ GeV. Finally, to suppress $Z\gamma \gamma$ contributions, events with $E_{\text {T}}^{\text {miss}}<50$ GeV are discarded. The event selection requirements for the $W\gamma \gamma$ CR are summarized in Table 1. A total of seven events are observed in this $W\gamma \gamma$ control region, of which 1.6 are expected to arise from sources other than $W\gamma \gamma$ production. The MC expectation for the $W\gamma \gamma$ process is 1.9 events, leading to a $W\gamma \gamma$ scale factor of $2.9 \pm 1.4$, assuming that no GGM signal events contaminate the $W\gamma \gamma$ CR. This scale factor is consistent with that of the corresponding $\sqrt{s} =$ 8 TeV analysis [3], and is reconciled by a large and uncertain NLO correction to the $W\gamma \gamma$ production cross section that depends strongly upon the momentum of the $W\gamma \gamma$ system [52]. When setting limits on specific signal models, a simultaneous fit to the control region and the signal region is performed, allowing both the signal and $W\gamma \gamma$ contributions to float to their best-fit values.

Last, the irreducible background from $Z(\rightarrow \nu \nu )\gamma \gamma$ events, the only background without a data-derived normalization, is estimated with simulation and found to be 0.02 events. A ${\pm }100~\%$ uncertainty is conservatively applied to account for modelling uncertainties [53].

A summary of the background contributions to the signal region is presented in Table 2. The QCD background can be traced to a few hundredths of an event at high $E_{\text {T}}^{\text {miss}}$and high $m_{\mathrm {eff}}$, but no events are observed for either the diphoton Monte Carlo or the jet-faking-photon control sample when the full signal region requirements are applied. Relaxing the $m_{\mathrm {eff}}$ requirement, and using a conservative extrapolation of the expected QCD background as a function of $m_{\mathrm {eff}}$, the combined QCD background is estimated to be $0.05^{+0.20}_{-0.05}$ events. The estimate of the electron-faking-photon background is established by the presence of two $e\gamma$ events in the background model passing the SR requirements, yielding a background estimate of $0.03 \pm 0.02$ events after application of the fake-factor weights. Summing all background contributions, a total of $0.27^{+0.22}_{-0.10}$ SM events are expected in the SR, with the largest contribution, $0.17 \pm 0.08$ events, expected to arise from $W\gamma \gamma$ production. The background modelling was found to agree well in several validation regions, including the inclusive high-$p_{\text {T}}$ diphoton sample, as well as event selections with relaxed $m_{\mathrm {eff}}$ and $E_{\text {T}}^{\text {miss}}$ requirements relative to those of the SR.

**Table 2:** Summary of background estimates by source, and total combined background, in the signal region. The uncertainties shown include the total statistical and systematic uncertainty. Also shown is the expected number of signal events for the benchmark points $(m_{\tilde{g}},m_{\tilde{\chi }^{0}_{1}}) = (1500,100)$ and $(m_{\tilde{g}},m_{\tilde{\chi }^{0}_{1}}) = (1500,1300)$, where all masses are in GeV

Source	Number of events
QCD ($\gamma \gamma$, $\gamma$j, jj)	$0.05^{+0.20}_{-0.05}$
$e\rightarrow \gamma$ fakes	$0.03 \pm 0.02$
$W\gamma \gamma$	$0.17 \pm 0.08$
$Z\gamma \gamma$	$0.02 \pm 0.02$
Sum	$0.27^{+ 0.22}_{- 0.10}$
$(m_{\tilde{g}},m_{\tilde{\chi }^{0}_{1}}) = (1500,100)$	7.0
$(m_{\tilde{g}},m_{\tilde{\chi }^{0}_{1}}) = (1500,1300)$	8.0

## Signal efficiencies and uncertainties

GGM signal acceptances and efficiencies are estimated using MC simulation for each simulated point in the gluino–bino parameter space, and vary significantly across this space due to variations in the photon $p_{\text {T}}$, $E_{\text {T}}^{\text {miss}}$, and $m_{\mathrm {eff}}$ spectra. For example, for a gluino mass of 1600 GeV, the acceptance-times-efficiency product varies between 14 and 28 %, reaching a minimum as the NLSP mass approaches the *Z* boson mass, below which the photonic branching fraction of the NLSP rises to unity. Table 3 summarizes the contributions to the systematic uncertainty of the signal acceptance-times-efficiency, which are discussed below.

Making use of a bootstrap method [54], the efficiency of the diphoton trigger is determined to be greater than 99 %, with an uncertainty of less than 1 %. The uncertainty in the integrated luminosity is ${\pm }2.1~\%$. It is derived, following a methodology similar to that detailed in Ref. [55], from a calibration of the luminosity scale using x–y beam-separation scans performed in August 2015.

The reconstruction and identification efficiency for tight, isolated photons is estimated with complementary data-driven methods [42]. Photons selected kinematically as originating from radiative decays of a *Z* boson ($Z \rightarrow \ell ^+ \ell ^- \gamma$ events) are used to study the photon reconstruction efficiency as a function of $p_{\text {T}}$ and $\eta$. Independent measurements making use of a tag-and-probe approach with $Z \rightarrow ee$ events, with one of the electrons used to probe the calorimeter response to electromagnetic depositions, also provide information about the photon reconstruction efficiency. For photons with $p_{\text {T}}> 75$ GeV, the identification efficiency varies between 93 and 99 %, depending on the values of the photon $p_{\text {T}}$ and $|\eta |$ and whether the photon converted in the inner detector. The uncertainty also depends upon these factors, and is generally no more than a few percent.

Uncertainties in the photon and jet energy scales lead to uncertainties in the signal acceptance-times-efficiency that vary across the GGM parameter space, and contribute the dominant source of acceptance-times-efficiency uncertainty in certain regions of the parameter space. The photon energy scale is determined using samples of $Z \rightarrow ee$ and $J/\psi \rightarrow ee$ events [56]. The jet-energy scale uncertainty is constrained from an assessment of the effect of uncertainties in the modelling of jet properties and by varying the response to differing jet flavour composition in MC simulations, as well as from *in situ* measurements with 8 TeV dijet data [46, 47].

Uncertainties in the values of whole-event observables, such as $E_{\text {T}}^{\text {miss}}$ and $m_{\mathrm {eff}}$, arise from uncertainties in the energy of the underlying objects from which they are constructed. Uncertainties in the $E_{\text {T}}^{\text {miss}}$ soft term due to uncertainties in hadronic fragmentation, detector material modeling and energy scale were found to introduce an uncertainty of less than 0.1 % in the signal acceptance-times-efficiency. The uncertainty due to pile-up is estimated by varying the mean of the distribution of the number of interactions per bunch crossing overlaid in the simulation by ${\pm }11~\%$.

Including the contribution from the statistical limitations of the MC samples used to model the GGM parameter space, the quadrature sum of the individual systematic uncertainties in the signal reconstruction efficiency is, on average, about 4 %. Adding the uncertainty in the integrated luminosity gives a total systematic uncertainty of about 5 %.

**Table 3:** Summary of individual and total contributions to the systematic uncertainty of the signal acceptance-times-efficiency. Relative uncertainties are shown in percent, and as the average over the full range of the ($m_{\tilde{g}}$,$m_{\tilde{\chi }^{0}_{1}}$) grid. Because the individual contributions are averaged over the grid only for that particular source, the average total uncertainty is not exactly equal to the quadrature sum of the individual average uncertainties

Source of systematic uncertainty	Value
Luminosity (%)	2.1
Photon identification (%)	3.0
Photon energy scale (%)	0.2
Photon energy resolution (%)	0.2
Jet energy scale (%)	0.4
Jet energy resolution (%)	0.3
$E_{\text {T}}^{\text {miss}}$ soft term (%)	${<}0.1$
Pile-up uncertainty (%)	1.8
MC statistics (%)	2.3
Total experimental uncert (%)	4.7

## Results

An accounting of the numbers of events observed in the SR after the successive application of the selection requirements is shown in Table 4 along with the size of the expected SM background. After the full selection is applied, no events are observed in the SR, to be compared to an expectation of $0.27^{+ 0.22}_{-0.10}$ SM events.

**Table 4:** Numbers of events observed in the SR after the successive application of the selection requirements, as well as the size of the expected SM background

Requirement	Number of events
Two photons, $p_{\mathrm {T}}^{\gamma }> 75$	4982
$\Delta \phi _{\mathrm {min}}(\mathrm {jet},\varvec{p}_{\mathrm {T}}^\mathrm{miss}{})> 0.5$	4724
$m_{\mathrm {eff}}> 1500$ GeV	1
$E_{\text {T}}^{\text {miss}}> 175$ GeV	0
Expected SM background	$0.27^{+ 0.22}_{- 0.10}$

Based on the observation of zero events in the SR and the magnitude of the estimated SM background expectation and uncertainty, an upper limit is set on the number of events from any scenario of physics beyond the SM, using the profile likelihood and $CL_s$ prescriptions [57]. The various sources of experimental uncertainty, including those in the background expectation, are treated as Gaussian-distributed nuisance parameters in the likelihood definition. Assuming that no events due to physical processes beyond those of the SM populate the $\ell \gamma \gamma$ CR used to estimate the $W(\rightarrow \ell \nu )+\gamma \gamma$ background, the observed 95 % confidence-level (CL) upper limit on the number of non-SM events in the SR is found to be 3.0. Taking into account the integrated luminosity of $3.2{~\mathrm{fb}^{-1}}$, this number-of-event limit translates into a 95 % CL upper limit on the visible cross section for new physics, defined by the product of cross section, branching fraction, acceptance and efficiency, of 0.93 fb.

**Fig. 3:**
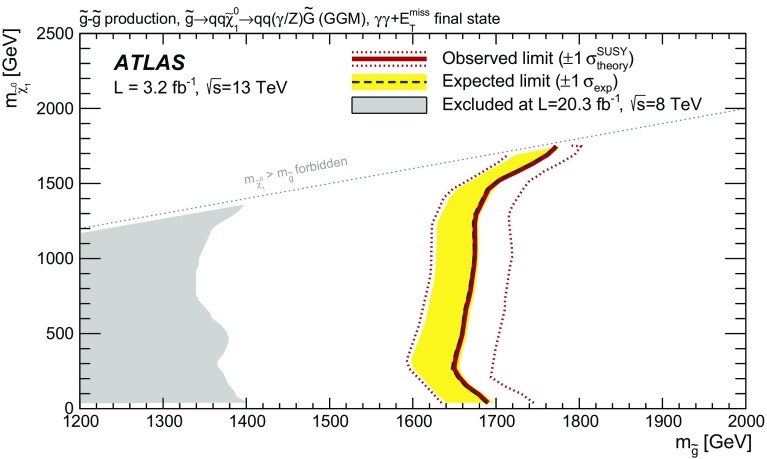
Exclusion limits in the neutralino–gluino mass plane at 95 % CL. The observed limits are exhibited for the nominal SUSY model cross section, as well as for a SUSY cross section increased and lowered by one standard deviation of the cross-section systematic uncertainty. Also shown is the expected limit, as well as the ${\pm }1$ standard-deviation range of the expected limit, which is asymmetric because of the low count expected. Because the background expectation is close to zero and the observed number of events is zero, the expected and observed limits nearly overlap. The previous limit from ATLAS using 8 TeV data [3] is shown in grey. Within the context of this model, gluino masses as low as 400 GeV have been excluded in a prior analysis making use of 7 TeV ATLAS data [58]

By considering, in addition, the value and uncertainty of the acceptance-times-efficiency of the selection requirements associated with the SR, as well as the NLO (+NLL) GGM cross Sect. [20–24], which varies steeply with gluino mass, 95 % CL lower limits may be set on the mass of the gluino as a function of the mass of the lighter bino-like neutralino, in the context of the GGM scenario described in Sect. 1. The resulting observed limit on the gluino mass is exhibited, as a function of neutralino mass, in Fig. 3. For the purpose of establishing these model-dependent limits, the $W(\rightarrow \ell \nu )+\gamma \gamma$background estimate and the limit on the possible number of events from new physics are extracted from a simultaneous fit to the SR and $W(\rightarrow \ell \nu )+\gamma \gamma$control region, although for a gluino mass in the range of the observed limit the signal contamination in the $W(\rightarrow \ell \nu )+\gamma \gamma$control sample is less than 0.03 events for any value of the neutralino mass. Also shown for this figure is the expected limit, including its statistical and background uncertainty range, as well as observed limits for a SUSY model cross section ${\pm }1$ standard deviation of theoretical uncertainty from its central value. Because the background expectation is close to zero and no events are observed in data, the expected and observed limits nearly overlap. The observed lower limit on the gluino mass is observed to be roughly independent of neutralino mass, reaching a minimum value of approximately 1650 GeV at a neutralino mass of 250 GeV. Within the context of this model, gluino masses as low as 400 GeV have been excluded in a prior analysis making use of 7 TeV ATLAS data [58].

## Conclusion

A search has been made for a diphoton + $E_{\text {T}}^{\text {miss}}$ final state using the ATLAS detector at the Large Hadron Collider in $3.2{~\mathrm{fb}^{-1}}$ of proton–proton collision data taken at a centre-of-mass energy of 13 TeV in 2015. At least two photon candidates with $p_{\text {T}}> 75~\text{GeV}$ are required, as well as minimum values of 175 and 1500 GeV of the missing transverse momentum and effective mass of the event, respectively. The resulting signal region targets events with pair-produced high-mass gluinos each decaying to either a high-mass or low-mass bino-like neutralino. Using a combination of data-driven and direct Monte Carlo approaches, the SM background is estimated to be $0.27^{+0.22}_{-0.10}$ events, with most of the expected background arising from the production of a *W* boson in association with two energetic photons. No events are observed in the signal region; considering the expected background and its uncertainty, this observation implies model-independent 95 % CL upper limits of 3.0 events (0.93 fb) on the number of events (visible cross section) due to physics beyond the Standard Model. In the context of a generalized model of gauge-mediated supersymmetry breaking with a bino-like NLSP, this leads to a lower limit of 1650 GeV on the mass of a degenerate octet of gluino states, independent of the mass of the lighter bino-like neutralino. This extends the corresponding limit of 1340 GeV derived from a similar analysis of 8 TeV data by the ATLAS Collaboration.
